# Supplementary material for: Potentiation of neuronal activity by tonic GluD1 current in brain slices
Source: EMBO Rep. 2023 May 8;24(7):e56801. doi: 10.15252/embr.202356801 (PMC10328076; doi:10.15252/embr.202356801)
Supplement: Supplementary file 1 — Expanded View Figures PDF [file EMBR-24-e56801-s008.pdf]

Expanded View Figures

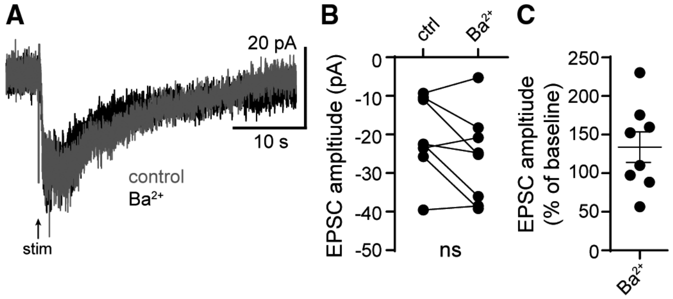

Figure EV1. External Ba^{2+} has no effect on the α1-adrenergic receptor-dependent excitatory postsynaptic current.

A Representative traces of electrically evoked (arrow) α1-A_R-EPSCs in control conditions and after application of Ba^{2+} (100 μM).

B Plot of the amplitude of the α1-A_R-EPSC in control conditions (ctrl) and after application of Ba^{2+} ($P = 0.20$, $n = 8$ biological replicates).

C Plot of the percent change in α1-A_R-EPSC amplitude with external Ba^{2+} ($n = 8$ biological replicates).

Data information: In (B, C), line and error bars represent mean ± SEM. ns denotes not significant (Wilcoxon matched-pairs signed rank test).
Source data are available online for this figure.

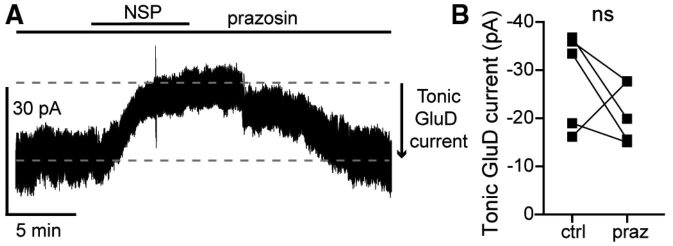

Figure EV2. Inverse agonism of α1-adrenergic receptors has no effect on tonic $GluD1_R$ current.

A Representative whole-cell voltage-clamp recording of the apparent outward current produced by application of NASPM (NSP) in the presence of an α1-adrenergic receptor inverse agonist, prazosin (100 nM).

B Plot of the magnitude of $GluD1_R$ tonic current in control conditions (ctrl) and after application of prazosin (praz; $P = 0.31$, $n = 5$ biological replicates).

Data information: In (B), ns denotes not significant (Wilcoxon matched-pairs signed rank test).
Source data are available online for this figure.

© 2023 The Authors

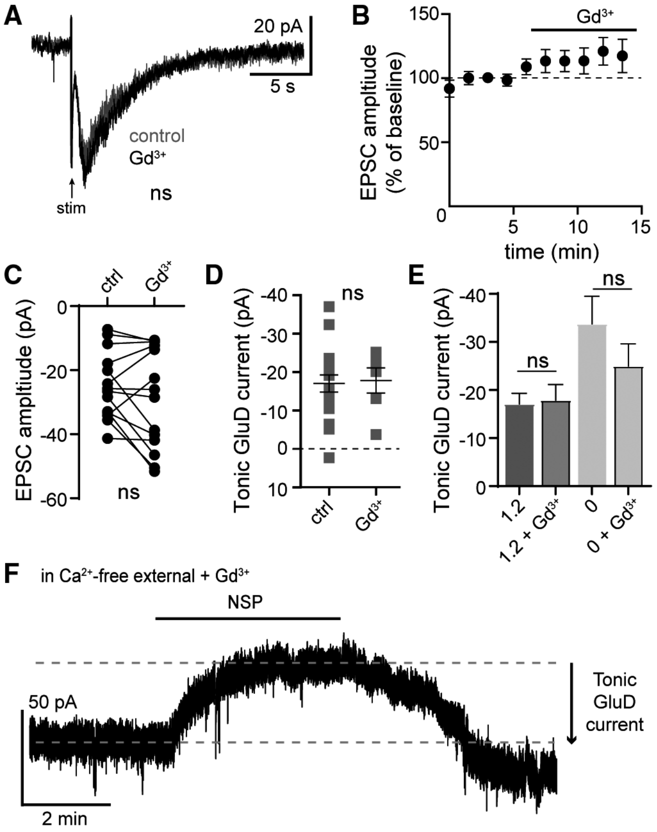

Figure EV3. GluD1$_R$ current is insensitive to gadolinium.

A Representative traces of electrically evoked (arrow) α1-A$_R$-EPSCs in control conditions and after application of Gd^{3+} (10 μM).

B Gd^{3+} had no significant effect on the amplitude of the α1-A$_R$-EPSC, shown in a time-course plot. Dashed line indicates 100% of baseline amplitude (n = 14 biological replicates).

C Plot of the amplitude of the α1-A$_R$-EPSC in control conditions (ctrl) and in Gd^{3+} (10 μM, $P = 0.15$, $n = 14$).

D Plot of the magnitude of tonic GluD1$_R$ current measured in control conditions (ctrl), or in the presence of Gd^{3+}, showing no difference in the amplitude of tonic GluD1$_R$ current ($P = 0.58$, n = 18 and 6 biological replicates).

E Plot of the magnitude of tonic GluD1$_R$ current measured in standard external calcium (1.2 mM) with and without Gd^{3+} (as shown in D) and in calcium-free external solution (0 mM) with and without Gd^{3+}. Gd^{3+} had no significant effect on the magnitude of the tonic GluD1$_R$ current (1.2 vs. 1.2+Gd^{3+}: $P > 0.999$; 1.2 vs. 0: $P = 0.024$; 1.2+Gd^{3+} vs. 0+Gd^{3+}: $P > 0.99$; 0 vs. 0+Gd^{3+}: $P > 0.99$, n = 18, 18, 6, and 14 biological replicates respectively).

F In nominally calcium-free external and Gd^{3+}, NASPM still produced a large apparent outward current, shown in a representative whole-cell voltage-clamp recording.

Data information: In (B–E) line and error bars represent mean ± SEM, ns denotes not significant (C: Wilcoxon test; D: Mann–Whitney test; E: Kruskal–Wallis test).
Source data are available online for this figure.

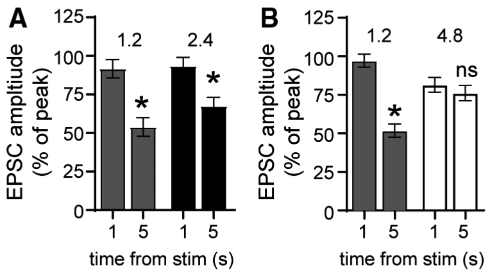

Figure EV4. High extracellular calcium prolongs the α1-adrenergic receptor-dependent excitatory postsynaptic current.

A Plot of the amplitude of the α1-A$_R$-EPSC relative to the peak amplitude, measured 1 and 5 s from stimulation in control conditions (1.2 mM calcium) and after application of 2.4 mM calcium. In both 1.2 and 2.4 mM calcium, the amplitude of the α1-A$_R$-EPSC is reduced significantly by 5 s after stimulation (1.2 mM, $P = 0.001$; 2.4 mM, $P = 0.017$, n = 14 biological replicates).

B Plot of the amplitude of the α1-A$_R$-EPSC relative to the peak amplitude, measured 1 and 5 s from stimulation in control conditions (1.2 mM calcium) and after application of 4.8 mM calcium. In 1.2 mM, but not in 4.8 mM calcium, the amplitude of the α1-A$_R$-EPSC is reduced significantly by 5 s after stimulation (1.2 mM, $P < 0.0001$; 4.8 mM, $P = 0.671$, n = 21 biological replicates).

Data information: Line and error bars represent mean ± SEM. * denotes statistical significance, ns denotes not significant: Two-way ANOVA tests.
Source data are available online for this figure.

© 2023 The Authors
