# Supplementary material for: Potentiation of neuronal activity by tonic GluD1 current in brain slices
Source: EMBO Rep. 2023 May 8;24(7):e56801. doi: 10.15252/embr.202356801 (PMC10328076; doi:10.15252/embr.202356801)
Supplement: Supplementary file 3 — PDF+ [file EMBR-24-e56801-s006.pdf]

Article

SOURCE DATA · TRANSPARENT PROCESS · OPEN ACCESS

EMBO reports

Potentiation of neuronal activity by tonic GluD1 current in brain slices

Daniel S Copeland, Aleigha Gugel & Stephanie C Gantz*

Abstract

Ion channel function of native delta glutamate receptors ($GluD_R$) is incompletely understood. Previously, we and others have shown that activation of Gαq protein-coupled receptors (GqPCR) produces a slow inward current carried by $GluD1_R$. $GluD1_R$ also carries a tonic cation current of unknown cause. Here, using voltage-clamp electrophysiological recordings from adult mouse brain slices containing the dorsal raphe nucleus, we find no role of ongoing G-protein-coupled receptor activity in generating or sustaining tonic $GluD1_R$ currents. Neither augmentation nor disruption of G protein activity affects tonic $GluD1_R$ currents, suggesting that ongoing G-protein-coupled receptor activity does not give rise to tonic $GluD1_R$ currents. Further, the tonic $GluD1_R$ current is unaffected by the addition of external glycine or D-serine, which influences $GluD2_R$ current at millimolar concentrations. Instead, GqPCR-stimulated and tonic $GluD1_R$ currents are regulated by physiological levels of external calcium. In current-clamp recordings, block of $GluD1_R$ channels hyperpolarizes the membrane by ~7 mV at subthreshold potentials, reducing excitability. Thus, $GluD1_R$ carries a G-protein-independent tonic current that contributes to subthreshold neuronal excitation in the dorsal raphe nucleus.

Keywords cation channel; delta glutamate; G protein; GluD; tonic current
Subject Category Neuroscience
DOI 10.15252/embr.202356801 | Received 9 January 2023 | Revised 19 April 2023 | Accepted 24 April 2023 | Published online 8 May 2023
EMBO Reports (2023) 24: e56801

Introduction

The majority of excitatory neurotransmission in the central nervous system is produced by ionic current carried by the ionotropic glutamate receptors (iGluRs). Lesser known in the iGluR family are the delta glutamate receptors ($GluD1_R$ and $GluD2_R$), which share < 30% amino acid sequence identity with the other family members (Araki *et al*, 1993; Lomeli *et al*, 1993). Either $GluD1_R$ or $GluD2_R$ is expressed in the central neurons in nearly every region of the adult mouse brain, with a high level of overlap at the regional and cellular level (Konno *et al*, 2014; Hepp *et al*, 2015; Nakamoto *et al*, 2020). Predominately, $GluD1_R$ and $GluD2_R$ are found in postsynaptic specializations on the dendrites and spines (Landsend *et al*, 1997; Hepp *et al*, 2015; Nakamoto *et al*, 2020; Hoover *et al*, 2021), where they regulate synapse formation, composition, and autophagy in complex with trans-synaptic and secreted proteins (Tao *et al*, 2018; Fossati *et al*, 2019; Dai *et al*, 2021; Gawande *et al*, 2021, 2022). The study of ion channel function of $GluD1_R$ has been limited since there is no known agonist that binds to $GluD1_R$ directly to gate opening of the channel. Nonetheless, we and others have demonstrated that $GluD1_R$ and $GluD2_R$ carry ionic current upon activation of $Gα_q$-protein-coupled receptors (GqPCRs), either metabotropic glutamate (mGluR, Ady *et al*, 2013; Dadak *et al*, 2017; Benamer *et al*, 2018) or α1-adrenergic receptors (Gantz *et al*, 2020), through a process that involves intact G protein signaling. Intriguingly, in cell lines and brain slices, $GluD1_R$ and $GluD2_R$ are open in the presumed absence of agonists and carry tonic cation current (Gantz *et al*, 2020; Lemoine *et al*, 2020). The cause of the tonic $GluD1_R$ current is unknown.

Typically, GPCRs are activated when extracellular ligands bind to the receptor and force a conformational change, which initiates downstream signal transduction mechanisms. In principle, GqPCRs could exhibit low levels of activation in response to ambient ligand, as demonstrated for $Gα_{i/o}$-protein-coupled dopamine D2 receptors (Rodriguez-Contreras *et al*, 2021). GPCRs can also be constitutively active, entering an active state conformation in the absence of ligand (reviewed in Bond & IJzerman, 2006). Despite knowledge that $GluD1_R$ is modulated by a GTP-dependent mechanism (Gantz *et al*, 2020), whether the tonic $GluD1_R$ current is a product of low-level GqPCR activity is not established.

Here, using patch-clamp electrophysiology in acute mouse brain slices, we show that inverse agonism of α1-adrenergic receptors ($α1\text{-}A_R$), which are capable of modulating $GluD1_R$ current, did not affect tonic $GluD1_R$ current, indicating that $α1\text{-}A_R$ activation was not responsible for generating tonic $GluD1_R$ current. Further, all methods employed to manipulate G protein activity did not impact the amplitude of the tonic $GluD1_R$ current. Thus, tonic $GluD1_R$ current arises from a mechanism separate from ongoing, cell-autonomous GPCR activity. Unlike recent observations with $GluD2_R$ (Carrillo *et al*, 2021), we find that $GluD1_R$ current was not affected by millimolar glycine or D-serine, making it unlikely that tonic $GluD1_R$ current arises from ambient levels of these amino acids. Instead, both GqPCR-stimulated and tonic $GluD1_R$ currents were regulated by physiological levels of extracellular calcium. Increasing

Department of Molecular Physiology and Biophysics, University of Iowa, Iowa City, IA, USA
*Corresponding author. Tel: +1 319 335 0541; E-mail: stephanie-gantz@uiowa.edu

© 2023 The Authors. Published under the terms of the CC BY 4.0 license.

extracellular calcium above 2 mM, which is higher than physiological levels but commonly found in artificial cerebral spinal fluids for *in vitro* research (Forsberg *et al*, 2019; Lopes & Cunha, 2019), reduced $GluD1_R$ unitary current and the magnitude of tonic $GluD1_R$ by ~50%. When measured at physiological levels of calcium, tonic $GluD1_R$ current contributes to subthreshold depolarization that drives action potential firing of dorsal raphe neurons.

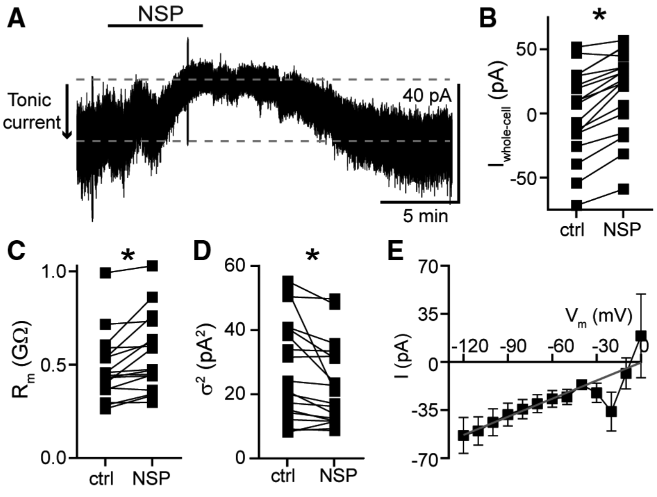

Figure 1. NASPM reveals a tonic inward current carried by $GluD1_R$.

A Representative whole-cell voltage-clamp recording of the apparent outward current produced by application of NASPM (NSP, 100 μM). Dashed lines indicate baseline current (bottom) and the peak of the outward current (top). Tonic current was measured as the difference of these lines (arrow).

B Plot of the whole-cell current (V_{hold} −65 mV) in control conditions (ctrl) and after application of NASPM (NSP, $P < 0.0001$, $n = 18$).

C Plot of basal membrane resistance recorded in control conditions (ctrl) and during NASPM application (NSP, $P = 0.0002$, $n = 17$).

D Plot of membrane noise variance (σ^2) in control conditions (ctrl) and during NASPM application (NSP, $P = 0.002$, $n = 18$).

E Current–voltage relationship of the NASPM-sensitive tonic inward current. The linear portion was fit by linear regression (gray line) indicating an E_{rev} near 0 mV ($n = 5$ biological replicates).

Data information: In (B–E), line and error bars represent mean ± SEM. In (B–D), * denotes statistical significance (Wilcoxon matched-pairs signed rank tests).
Source data are available online for this figure.

Results

$GluD1_R$ carries a tonic current

Whole-cell voltage-clamp recordings were made from dorsal raphe neurons in acute brain slices from wild-type mice at 35°C in the presence of GluN, GluA, and GluK receptor blockers, using a potassium-based internal solution (V_{hold} − 65 mV). Our previous work showed that $GluD1_R$ carries an ~−20 pA tonic current, revealed by the application of a channel blocker, 1-naphthyl acetyl spermine (NASPM) and by genetic deletion of $GluD1_R$ (Gantz *et al*, 2020). In agreement with our prior work, here we show that application of NASPM (100 μM) produced an apparent outward current of 17.1 ± 2.2 pA (Fig 1A and B). On average, the current peaked in 3.5 min and reversed in 11 min upon washout of NASPM. The outward current was accompanied by an increase in the membrane resistance (Fig 1C) and a reduction in the membrane noise variance (σ^2, Fig 1D), indicating fewer open channels. NASPM failed to change the current when external Na^+ (126 mM) was replaced with N-methyl D-glucamine (−3.0 ± 3.6 pA, $P = 0.50$, $n = 3$). Voltage ramps from −120 to 10 mV (1 mV/10 ms) before and after application of NASPM showed that tonic $GluD1_R$ current reversed polarity at ~−2 mV (Fig 1E). In all, these findings reproduce those of our previous work (Gantz *et al*, 2020) and demonstrate that NASPM blocks a tonic, sodium-dependent inward current carried by $GluD1_R$.

Tonic $GluD1_R$ current is not produced by cell-autonomous G protein activity

$GluD1_R$ and $GluD2_R$ carry ionic current following the activation of either mGluR or α1-A_R (Ady *et al*, 2013; Dadak *et al*, 2017; Benamer *et al*, 2018; Gantz *et al*, 2020; Lemoine *et al*, 2020) via a G-protein-dependent mechanism (Dadak *et al*, 2017; Gantz *et al*, 2020). GPCRs can exhibit constitutive activity in the absence of agonist (Prézeau *et al*, 1996), and low-level constitutive activity of GPCRs affects other subthreshold cation conductances (Lu *et al*, 2010; Shen *et al*, 2012; Zhang *et al*, 2012; Quallo *et al*, 2017; Philippart & Khaliq, 2018). But the involvement of GqPCRs in generating tonic $GluD1_R$ current has not been explored.

To test whether increased G protein activity was sufficient for generating tonic $GluD1_R$ current, the internal recording solution was supplemented with a non-hydrolyzable GTP analog (guanosine-5′-[(β, γ)-imido]triphosphate, GppNHp, 1 mM), which binds irreversibly to Gα and elevates G protein activity. In dorsal raphe neurons, dialysis with GppNHp produces a tonic outward current carried by G protein-coupled inwardly rectifying potassium (GIRK) channels (Loucif *et al*, 2006) by elevating free Gβγ subunits, which gate GIRK channels (Pfaffinger *et al*, 1985). In agreement, whole-cell dialysis of GppNHp-containing internal solution (≥ 10 min) produced a tonic outward current with a reversal potential of ~−108 mV (Fig 2A–C), consistent with the expected reversal potential of potassium (calculated E_K: −104 mV). Application of $BaCl_2$ (100 μM), which blocks GIRK channels (Gantz *et al*, 2013), produced an apparent inward current (−46.0 ± 10.6 pA, Fig 2A) with GppNHp- but not GTP-containing internal solution (Fig 2C). These data demonstrate that amplifying G protein signaling with GppNHp produces standing currents carried by G protein-gated ion channels, consistent with previous studies (Loucif *et al*, 2006; Kramer & Williams, 2016). In the continued presence of $BaCl_2$, tonic $GluD1_R$ current was measured following application of NASPM (Fig 2D and E). On average, tonic $GluD1_R$ current was −16.7 ± 2.8 pA, which was not different from current measured with GTP-containing internal solution (Fig 2D). To determine whether $BaCl_2$ affected conductance of $GluD1_R$, we also examined $GluD1_R$ current stimulated by synaptic activation of α1-A_R (Gantz *et al*, 2020; Khamma *et al*, 2022). Electrical stimulation of the brain slice (5 pulses, 0.5 ms, 60 Hz) delivered via a monopolar stimulating electrode was used to evoke an α1-A_R-dependent excitatory postsynaptic current (α1-A_R-EPSC), which is carried by $GluD1_R$ (Gantz *et al*, 2020). External $BaCl_2$ (100 μM) had no effect on the amplitude of

 © 2023 The Authors

the α1-A_R-EPSC (ctrl: -20.6 ± 3.6 pA; $BaCl_2$: -26.0 ± 4.1 pA, Fig EV1A–C), indicating that at this concentration, external $BaCl_2$ does not affect conductance of $GluD1_R$. Taken together, the data suggest that augmenting G protein activity has a negligible impact on the amplitude of the tonic current carried by $GluD1_R$.

In principle, GPCRs could be activated by ambient ligand in brain slices to produce a small tonic current. In midbrain dopamine neurons, ambient activation of $G\alpha_{i/o}$-coupled dopamine D2 receptors produces a tonic GIRK current of ~9 pA (Rodriguez-Contreras *et al*, 2021). Next, we tested whether tonic $GluD1_R$ current was dependent on α1-A_R activity, either from ambient ligand or constitutive activity, by applying an α1-A_R inverse agonist prazosin (100 nM, Hein *et al*, 2001). Prazosin had no effect on the magnitude of the tonic $GluD1_R$ current (-19.9 ± 2.8 pA, Fig EV2A and B). In midbrain dopamine neurons, $GluD1_R$ current is produced by activation of mGluR (Benamer *et al*, 2018), suggesting that a similar mechanism may occur in the dorsal raphe. Moreover, in the dorsal raphe, $G\alpha_q$-coupled histamine H_1 and orexin OX_2 receptors converge on the same downstream effectors as α1-A_R (Brown *et al*, 2002), suggesting that if tonic $GluD1_R$ current was produced via G protein activity, there are many types of receptors to consider. As a broad test as to whether tonic $GluD1_R$ current was dependent on G protein signaling, recordings were made with an internal solution where GTP was replaced with a non-hydrolyzable analog of GDP, GDPβS-Li_3 (1.24 mM), which acts as a competitive antagonist at GTP-binding sites and arrests G protein signaling. Within 10 min of whole-cell dialysis with GDPβS-containing internal solution, application of noradrenaline (30 μM) produced an inward $GluD1_R$ current (Fig 3A and B). By ≥ 20 min of whole-cell dialysis, the noradrenaline-induced current was abolished (Fig 3A and B), confirming efficacy of GDPβS to arrest α1-A_R-$GluD1_R$ signaling (Gantz *et al*, 2020). In contrast, GDPβS had no effect on tonic $GluD1_R$ current when compared with GTP-containing internal solution with or without a similar concentration of LiCl (GDPβS-Li_3: -28.7 ± 4.9 pA, $n = 13$, GTP: -19.3 ± 2.7 pA, GTP + LiCl: -23.5 ± 3.9 pA, Fig 3C). Further, there was no decrement in the magnitude of tonic $GluD1_R$ current with repeat applications of NASPM (Fig 3D and E). These results demonstrate that tonic $GluD1_R$ current is independent of cell-autonomous G protein signaling.

Collectively we have shown, using pharmacological and genetics strategies, that $GluD1_R$ carries ionic current that can be observed in two ways: either by activating α1-A_R, which augments $GluD1_R$ current in a G-protein-dependent manner, or by measuring tonic $GluD1_R$ current that arises from a G-protein-independent mechanism. These features distinguish $GluD1_R$ current from sodium current carried by sodium leak NALCN channels. NALCN current is stimulated by GqPCR activation in a G-protein-independent manner (Lu *et al*, 2009), whereas "tonic" NALCN current is G-protein-dependent (Lu *et al*, 2010; Philippart & Khaliq, 2018; reviewed in Ren, 2011). Nonetheless, sufficient similarity between the conductances warrants closer examination of the pharmacological tools used on these channels. The trivalent ion gadolinium (Gd^{3+}) effectively blocks NALCN channels, with > 80% reduction in NALCN current with 10 μM Gd^{3+} (Lu *et al*, 2007, 2009, 2010; Chua *et al*, 2020). Here, Gd^{3+} (10 μM) had no significant effect on the amplitude of the α1-A_R-EPSC (Fig EV3A–C) nor on the magnitude of the tonic NASPM-sensitive current (Fig EV3D and E).

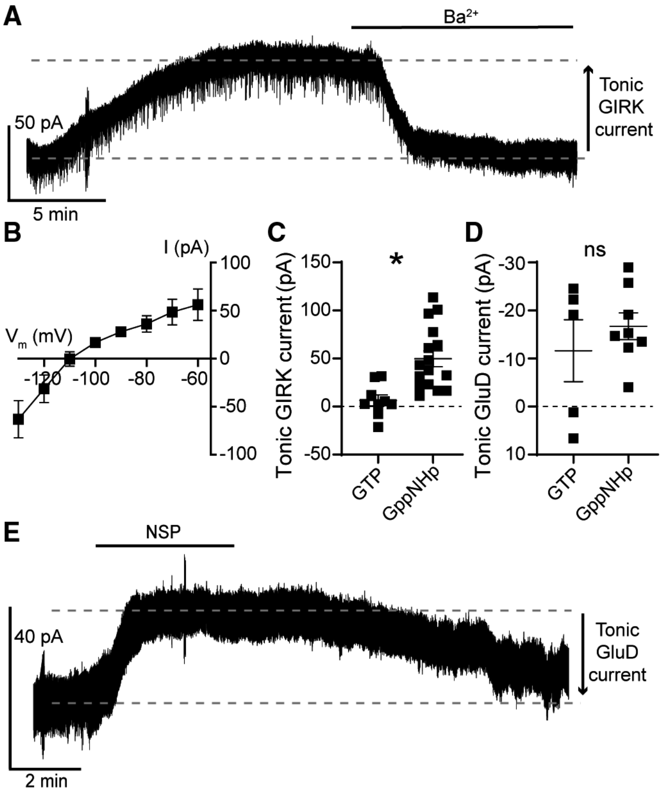

Figure 2. Augmentation of G protein activity with GppNHp has no effect on tonic $GluD1_R$ current.

A GppNHp-containing internal solution produced a tonic Ba^{2+}-sensitive (100 μM) outward current carried by GIRK channels, shown in a representative whole-cell voltage-clamp recording.

B Current–voltage relationship of the tonic Ba^{2+}-sensitive (100 μM) outward GIRK current demonstrating reversal near expected E_K and inward rectification ($n = 6$ biological replicates).

C Plot of tonic GIRK currents measured in control conditions (GTP) and with GppNHp-containing internal solution ($P = 0.0003$, $n = 9$ and 16 biological replicates respectively).

D Plot of the magnitude of tonic $GluD1_R$ current measured with GTP-containing internal solution as compared to GppNHp-containing internal solution ($P = 0.72$, $n = 5$ and 8 biological replicates respectively) when measured in external Ba^{2+} to block tonic GIRK current.

E GppNHp had no effect on the NASPM (NSP, 100 μM)-sensitive inward current, shown in a representative trace.

Data information: In (B–D), line and error bars represent mean ± SEM.
* denotes statistical significance, ns denotes not significant (Mann–Whitney tests).
Source data are available online for this figure.

Tonic $GluD1_R$ current is not augmented by glycine or D-serine

Using clusters of HEK-293T cells and synaptically connected cultured cerebellar neurons, Carrillo *et al* (2021) demonstrate that $GluD2_R$ can be opened by external glycine or D-serine with an EC_{50} of ~1 and 3 mM respectively and produce a steady-state current. Previously, we demonstrated that high concentrations (10 mM) of glycine and D-serine reduce the α1-A_R-EPSC without affecting unitary channel current (Gantz *et al*, 2020); attributing the diminished current to glycine inducing a desensitized state of the channel (Hansen *et al*, 2009). When applied at 1 mM, neither glycine (in the

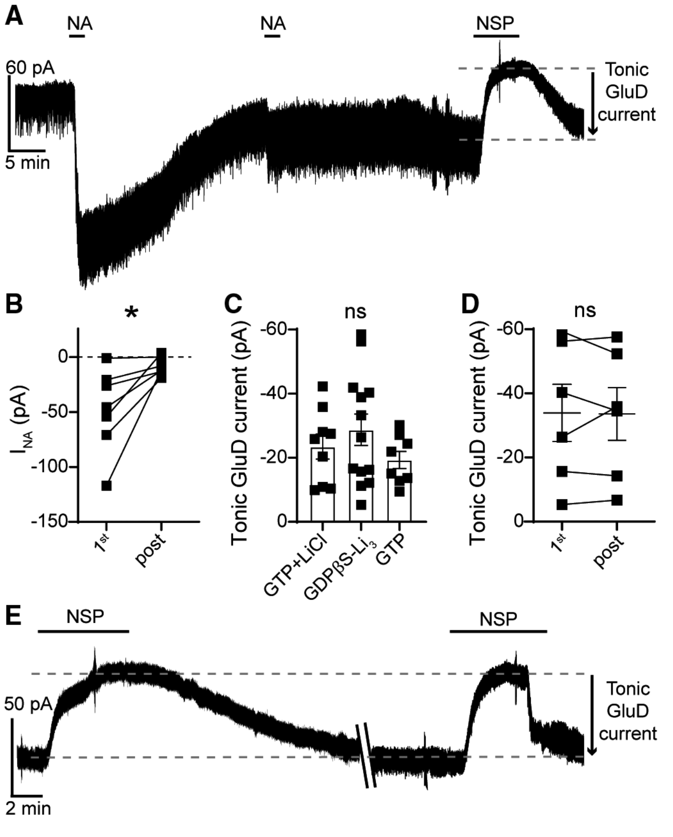

Figure 3. Tonic GluD1$_R$ current is not dependent on G protein signaling.

A With GDPβS-containing internal solution, noradrenaline-induced GluD1$_R$ current (I_{NA}) was diminished by > 20 min post-dialysis, shown in a representative trace.

B With GDPβS-containing internal solution, the amplitude of I_{NA} ran down with whole-cell dialysis; shown in a plot of the first application of noradrenaline (30 μM, 1st) compared to application of noradrenaline > 20 min post-dialysis (post, $P = 0.02$, $n = 7$).

C Plot of the magnitude of tonic GluD1$_R$ current measured after dialysis with GTP$^+$LiCl$^-$, GDPβS-Li$_3$-, and GTP-containing internal solution, displaying no significant difference between the groups ($P = 0.46$, $n = 9, 13, 8$ biological replicates respectively).

D Plot of the magnitude of tonic GluD1$_R$ current recorded with GDPβS-containing internal solution for the first application of NASPM (1st) and application of NASPM > 45 min post-dialysis, showing no difference in the average amplitude (post, $P = 0.84$, $n = 6$ biological replicates).

E With GDPβS-containing internal solution, repeated application of NASPM revealed tonic GluD1$_R$ current without a decrement in amplitude, shown in a representative trace. \\ indicate a 40-min wash in the recording.

Data information: In (B–D), line and error bars represent mean ± SEM. * denotes statistical significance, ns denotes not significant (B and D: Wilcoxon matched-pairs signed rank tests; C: Kruskal–Wallis test).
Source data are available online for this figure.

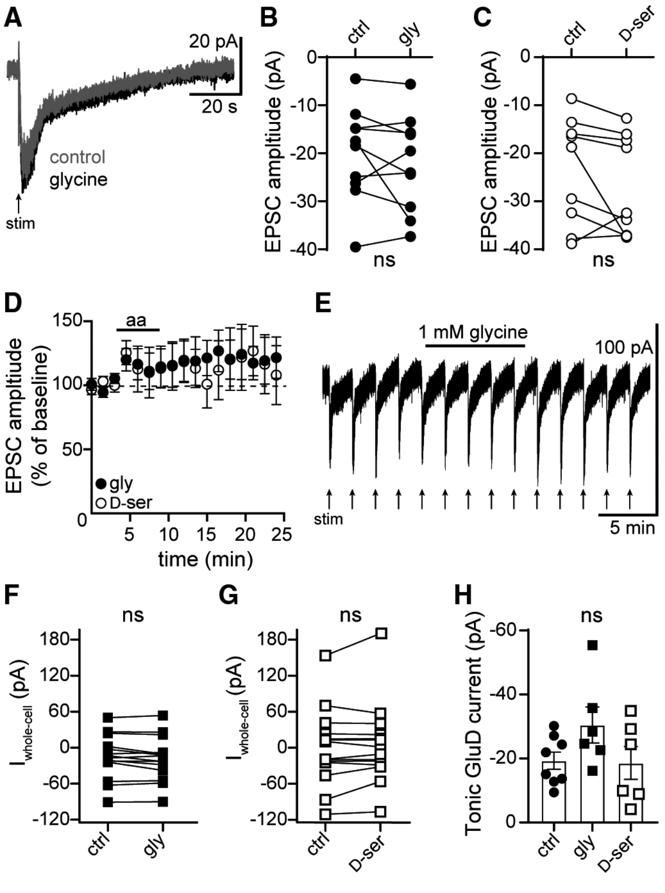

Figure 4. Tonic GluD1$_R$ current is not produced by external glycine or D-serine.

A Representative traces of electrically evoked (arrow) α1-A$_R$-EPSCs in control conditions and after application of glycine (1 mM).

B, C Plot of the amplitude of the α1-A$_R$-EPSC in control conditions (ctrl) and in glycine (1 mM, $P = 0.43$, $n = 10$ biological replicates, B), or D-serine (1 mM, $P = 0.13$, $n = 9$ biological replicates, C).

D Glycine or D-serine (aa) had no significant effect on the amplitude of the α1-A$_R$-EPSC, shown in a time-course plot ($n = 10$ and 9 biological replicates for glycine and D-serine respectively).

E Glycine had no significant effect on the amplitude of the α1-A$_R$-EPSC (arrow) or whole-cell current, shown in a representative trace.

F, G Plot of the whole-cell current in control conditions (ctrl) and in glycine ($P = 0.39$, $n = 14$ biological replicates, F) or D-serine ($P = 0.54$, $n = 14$ biological replicates, G).

H Plot of the magnitude of tonic GluD1$_R$ current measured in control conditions (ctrl), or in the presence of glycine (gly) or D-serine (D-ser), showing no difference in the amplitude of tonic GluD1$_R$ current ($P = 0.20$, $n = 8, 6, 6$ biological replicates respectively).

Data information: In (B–D and F–H), line and error bars represent mean ± SEM. ns denotes not significant (B, C and F, G: Wilcoxon matched-pairs signed rank tests; H: Kruskal–Wallis test).
Source data are available online for this figure.

presence of 10 μM strychnine) nor D-serine affected the α1-A$_R$-EPSC (Fig 4A–E). Neither glycine nor D-serine produced a change in whole-cell current (Fig 4E–G) or membrane resistance (glycine: $P = 0.72$, $n = 15$; D-serine: $P = 0.09$, $n = 12$). Lastly, the magnitude of tonic GluD1$_R$ current was measured by application of NASPM (100 μM) in the presence of glycine or D-serine (1 mM). Tonic GluD1$_R$ current was not changed by either amino acid at this concentration (Fig 4H).

One possibility is that glycine and D-serine do not directly gate GluD1$_R$, as observed for GluD2$_R$ (Carrillo *et al*, 2021). Alternatively, if tonic GluD1$_R$ current is produced by ambient levels of glycine or D-serine, the channels may be open in a desensitized low-conductance state, akin to steady-state current produced by conducting desensitized GluA$_R$ (Coombs *et al*, 2019). In

 © 2023 The Authors

constitutively open mutant $GluD2_R$, desensitization by D-serine is reduced dramatically by high levels of extracellular calcium (> 3 mM) through a mechanism that involves calcium binding and stabilization of the ligand-binding domain dimer interface (Hansen *et al*, 2009). Therefore, we increased the concentration of calcium in the extracellular solution to 4.8 mM and then applied glycine (1 mM). In 4.8 mM calcium, glycine (in the presence of 10 μM strychnine) had no effect on the $\alpha1$-A_R-EPSC (99.2 ± 5.5% of the amplitude in glycine) or whole-cell current (−0.42 ± 2.6 pA change in glycine, $P = 0.84$ for both comparisons, $n = 6$, Wilcoxon matched-pairs signed rank tests). Together these results suggest that $GluD1_R$ current is not affected by millimolar glycine or D-serine. Further it is unlikely that tonic $GluD1_R$ current arises from ambient levels of glycine or D-serine.

Tonic $GluD1_R$ current is reduced by elevated extracellular calcium

To further examine whether tonic $GluD1_R$ current is a product of desensitized low-conductance channels, we characterized the effect of increasing the concentration of extracellular calcium. Increasing extracellular calcium from physiological 1.2 mM (Forsberg *et al*, 2019, our standard recording solution) to 2.4 mM had no significant effect on the amplitude of the $\alpha1$-A_R-EPSC ($P = 0.18$, $n = 18$, Fig 5A), the time-to-peak ($P = 0.22$, $n = 14$), or the rate of decay ($P = 0.17$, $n = 14$, Wilcoxon matched-pairs signed rank tests). However, increasing extracellular calcium to 4.8 mM rapidly reduced the $\alpha1$-A_R-EPSC by ~40% (Fig 5A and B) accompanied by a significant slowing in the rate of decay (Fig 5B and C). Typically, the $\alpha1$-A_R-EPSC peaks in ~1 s from stimulation (Khamma *et al*, 2022) and decays by ~50% by 5 s from stimulation (Fig EV4A and B). In 4.8 mM calcium, the $\alpha1$-A_R-EPSC persisted with no significant decrement in amplitude for 5 s from stimulation (Fig EV4B). In 1.2 mM calcium, the $\alpha1$-A_R-EPSC membrane noise variance (σ^2)–amplitude relationship was fit well by linear regression, yielding an estimate of a −1.04 pA unitary current (Fig 5D), consistent with our previous report (Gantz *et al*, 2020). In 2.4 mM calcium, there was a significant decrease in the slope of the $\alpha1$-A_R-EPSC σ^2–amplitude relationship ($P = 0.044$, $n = 73$ and 20, simple linear regression) yielding an estimate of a −0.54 pA unitary current (Fig 5D). In 4.8 mM calcium, there was no longer a relationship between the $\alpha1$-A_R-EPSC membrane noise variance (σ^2) and amplitude (Fig 5D). Thus, elevating extracellular calcium reduces but prolongs the time course of $\alpha1$-A_R-stimulated $GluD1_R$ current.

Next, we assessed the magnitude of the tonic $GluD1_R$ current. On average, the tonic $GluD1_R$ current was −9.0 and −7.5 pA in 2.4 and 4.8 mM calcium, respectively (Fig 5E and F). When compared with measurements in 1.2 mM calcium, elevating extracellular calcium caused significant reductions in the magnitude of tonic $GluD1_R$ current (Fig 5F). To determine whether $GluD1_R$ current was reduced by resting levels of extracellular calcium, we measured tonic $GluD1_R$ current in nominally calcium-free external solution. Following the elimination of the $\alpha1$-A_R-EPSC, which served as a control for the removal of extracellular calcium (Gantz *et al*, 2020), NASPM was applied. On average, the magnitude of the tonic $GluD1_R$ current in nominally calcium-free solution was larger, ~−30 pA (Fig 5G and H). The magnitude of the tonic $GluD1_R$ current measured in nominally calcium-free solution was not changed significantly by the addition of the Gd^{3+} (10 μM, Fig EV3F).

In our prior work, we demonstrated that the $GluD1_{Rs}$ that underlie the $\alpha1$-A_R-EPSC are at least transiently open at rest since the channel pores were accessible to the open-channel blocker NASPM in the absence of $\alpha1$-A_R stimulation (Koike *et al*, 1997; Gantz *et al*, 2020). To determine whether the loss of $GluD1_R$ current in elevated extracellular calcium reflects a change in gating of $GluD1_R$, we tested whether increasing extracellular calcium affected the block of the $\alpha1$-A_R-EPSC by NASPM. Consistent with our prior work (Gantz *et al*, 2020), when applied in 1.2 mM calcium, NASPM (100 μM, 6 min) blocked the $\alpha1$-A_R-EPSC (93.3 ± 1.6% reduction, Fig 5I). In 2.4 mM calcium, NASPM blocked the $\alpha1$-A_R-EPSC to a similar degree (92.9 ± 2.0% reduction, Fig 5I). In 4.8 mM calcium, NASPM blocked $\alpha1$-A_R-EPSC, but to a lesser degree (80.9 ± 3.5% reduction, Fig 5I). These data indicate that in 4.8 mM calcium, $GluD1_R$ are less accessible to open-channel block by NASPM. In $GluN_R$, pore block by magnesium prevents binding of the open-channel blocker MK-801 (Reynolds & Miller, 1988; Hubbard *et al*, 1989). Since both magnesium and MK-801 block the pore and eliminate $GluN_R$ current, the "protection" provided by magnesium is apparent in the rate of recovery of the $GluN_R$ current upon washout and dissociation of MK-801 (McKay *et al*, 2013). Therefore, we examined the rate of recovery of the $\alpha1$-A_R-EPSC upon washout of NASPM. NASPM was applied in either 1.2 or 4.8 mM calcium, then NASPM was washed out and the $\alpha1$-A_R-EPSC recovered in 1.2 mM calcium (Fig 5J and K). After 12 min of wash, the $\alpha1$-A_R-EPSC blocked by NASPM in 4.8 mM calcium had fully recovered (105 ± 3.5%), whereas the $\alpha1$-A_R-EPSC blocked by NASPM in 1.2 mM calcium had only partially recovered (49.9 ± 10.0%, Fig 5K). Therefore, high levels of extracellular calcium inhibit $GluD1_R$ in a way that antagonizes open-channel block by NASPM. Taken together, the data suggest that extracellular calcium has direct inhibitory action on $GluD1_R$ current.

Tonic $GluD1_R$ current provides subthreshold drive of action potential firing

In vivo and *in vitro*, dorsal raphe neurons fire action potentials (APs) in a rhythmic "pacemaker" manner. Primarily, serotonin neurons are not autonomous pacemakers, but require subthreshold drive from noradrenergic afferents and activation of $\alpha1$-A_R (Baraban *et al*, 1978). In the absence of noradrenaline, dorsal raphe neurons are silent or fire slowly and erratically (Svensson *et al*, 1975; Baraban *et al*, 1978). To determine if tonic $GluD1_R$ current contributed to subthreshold excitation, whole-cell current clamp recordings were made from dorsal raphe neurons, and APs were evoked with somatic current injection (1.5 s, 20 pA increments, Fig 6A). Consistent with the absence of ambient noradrenaline in brain slices (Gantz *et al*, 2020), 5/10 neurons were firing spontaneously at a slow and irregular rate (0.8 ± 0.3 Hz); but all fired in response to current injection. After application of NASPM (30–50 μM), 1/10 neurons fired spontaneously, and the rest became quiescent (silent) until APs were evoked by current injection (Fig 6B). At subthreshold potentials (−80 to −55 mV), NASPM hyperpolarized the membrane by ~7–10 mV (Fig 6C), consistent with Ohm's law given the magnitude of the tonic $GluD1_R$ current (−17.1 ± 2.2 pA) and basal membrane resistance (481.1 ± 43.2 MΩs). Consequently, NASPM increased the minimum current necessary to evoke AP firing

© 2023 The Authors

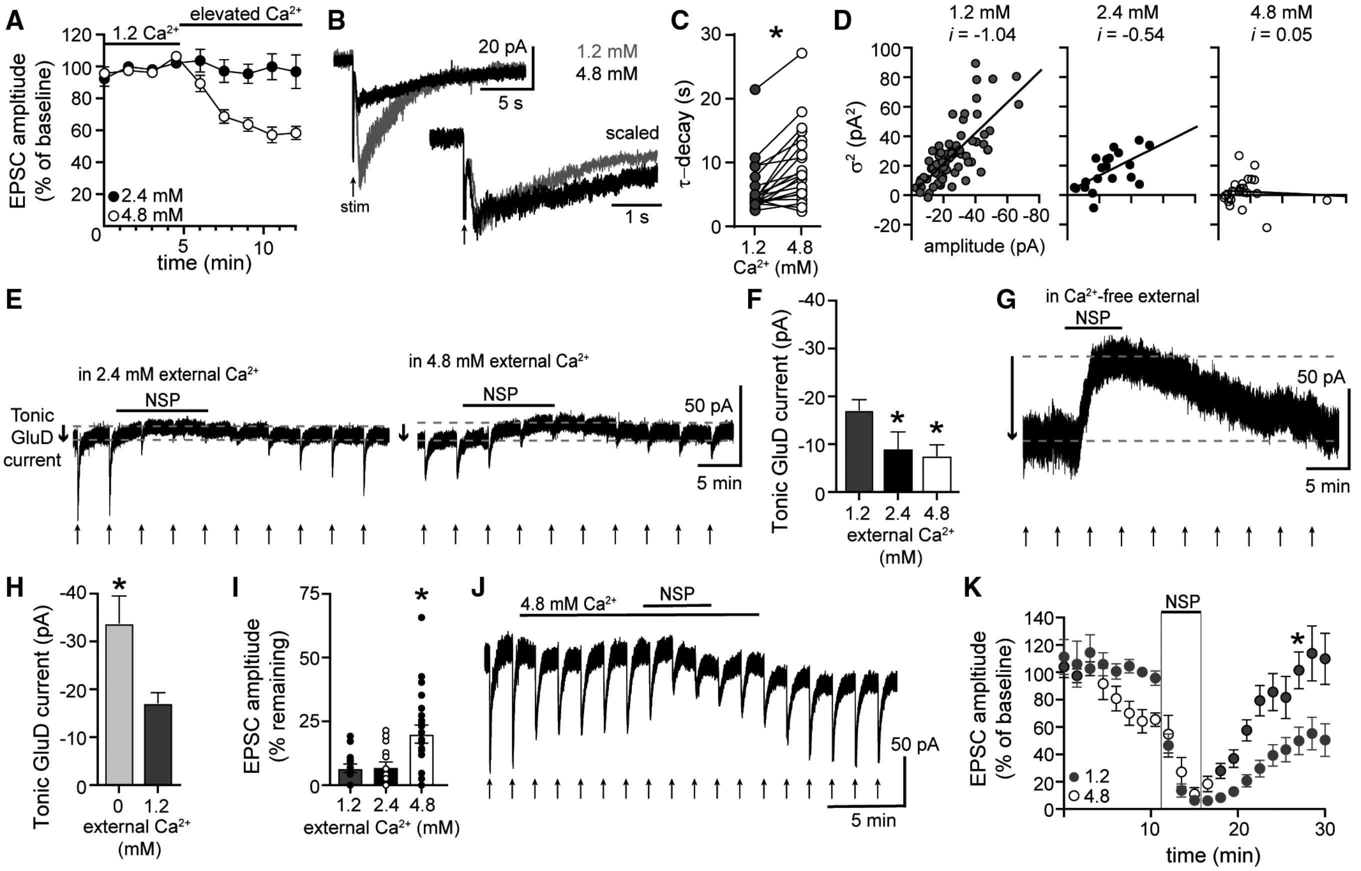

Figure 5. GluD1$_R$ current is inhibited by extracellular calcium.

A Increasing extracellular calcium to 4.8 mM reduced the amplitude of the α1-A_R-EPSC, shown in a time-course plot (n = 18 and 26 biological replicates for 2.4 and 4.8 mM respectively).

B Representative traces of electrically evoked (arrow) α1-A_R-EPSCs in control conditions (1.2 mM calcium) and after application of 4.8 mM calcium. Lower right inset shows the same traces scaled to their peak to illustrate the increase in the rate of decay in 4.8 mM calcium.

C Plot of the rate of decay of the α1-A_R-EPSC in control conditions (1.2 mM) and in 4.8 mM extracellular calcium (P < 0.0001, n = 21 biological replicates).

D Plots of the α1-A_R-EPSC variance versus mean amplitude in control conditions (1.2 mM), 2.4 mM, and 4.8 mM extracellular calcium, linear fits represent mean unitary current (i, 1.2 mM: r^2 = 0.53, P < 0.0001, n = 73; 2.4 mM: r^2 = 0.36, P = 0.006, n = 20; 4.8 mM: r^2 = 0.004, P = 0.748, n=26 biological replicates).

E Elevating extracellular calcium to 2.4 and 4.8 mM decreased the response to NASPM (NSP), shown in representative traces. Arrows indicate time of electrical stimulation and resulting α1-A_R-EPSCs.

F Plot of the magnitude of tonic GluD1$_R$ current measured by application of NASPM in control conditions (1.2 mM), 2.4 and 4.8 mM calcium. The magnitude of tonic GluD1$_R$ current in 2.4 and 4.8 mM calcium was reduced relative to control conditions (1.2 mM: n = 18; 2.4 mM: P = 0.03, n = 17; 4.8 mM: P = 0.005, n = 22 biological replicates).

G In nominally calcium-free external, NASPM produced a large apparent outward current, shown in a representative whole-cell voltage-clamp recording. Arrows indicate time of electrical stimulation and absence of α1-A_R-EPSCs.

H Plot of the magnitude of tonic GluD1$_R$ current measured by application of NASPM in control conditions (1.2 mM) and nominally calcium-free (0) solution. The magnitude of tonic GluD1$_R$ current in calcium-free was larger than control conditions (P = 0.009, n = 18 and 14 biological replicates).

I Plot of the inhibition of the α1-A_R-EPSC by application of NASPM in control conditions (1.2 mM), 2.4 mM, and 4.8 mM extracellular calcium (1.2 vs. 2.4 mM: P > 0.999, 1.2 vs. 4.8 mM: P = 0.026, n = 14, 15, and 22 biological replicates).

J Elevating extracellular calcium to 4.8 mM reduces the change in whole-cell current to NASPM and accelerates recovery of the α1-A_R-EPSC from NASPM-block in control conditions (1.2 mM extracellular calcium), shown in a representative whole-cell voltage-clamp recording.

K Time course of the block and recovery of the α1-A_R-EPSC by application of NASPM when applied in 4.8 mM extracellular calcium but allowed to recover in control conditions (1.2 mM), shown in comparison with the time course of the block and recovery of the α1-A_R-EPSC by application of NASPM when applied in control conditions (1.2 mM extracellular, as shown in C). Gray fill circles indicate measurements made in 1.2 mM, whereas open circles indicate measurements made in 4.8 mM extracellular calcium (P = 0.0007, 1.2: n = 7–14, 4.8: n = 6–7 biological replicates).

Data information: In (A, F, H, I, K), line and error bars represent mean ± SEM. * denotes statistical significance, ns denotes not significant (C: Wilcoxon matched-pairs signed rank test; D: Simple linear regression; F and I: Kruskal–Wallis tests; H and K: Mann–Whitney tests).
Source data are available online for this figure.

(approximate rheobase), which reversed upon 10 min washout of NASPM (Fig 6D) and increased the latency to fire the first AP (Fig 6E). In contrast, once the membrane reached threshold, NASPM had little-to-no effect on average membrane potential between APs (Fig 6C). Further, the AP waveform was unaffected by NASPM (Fig 6F). There were no differences in the AP half-width

 © 2023 The Authors

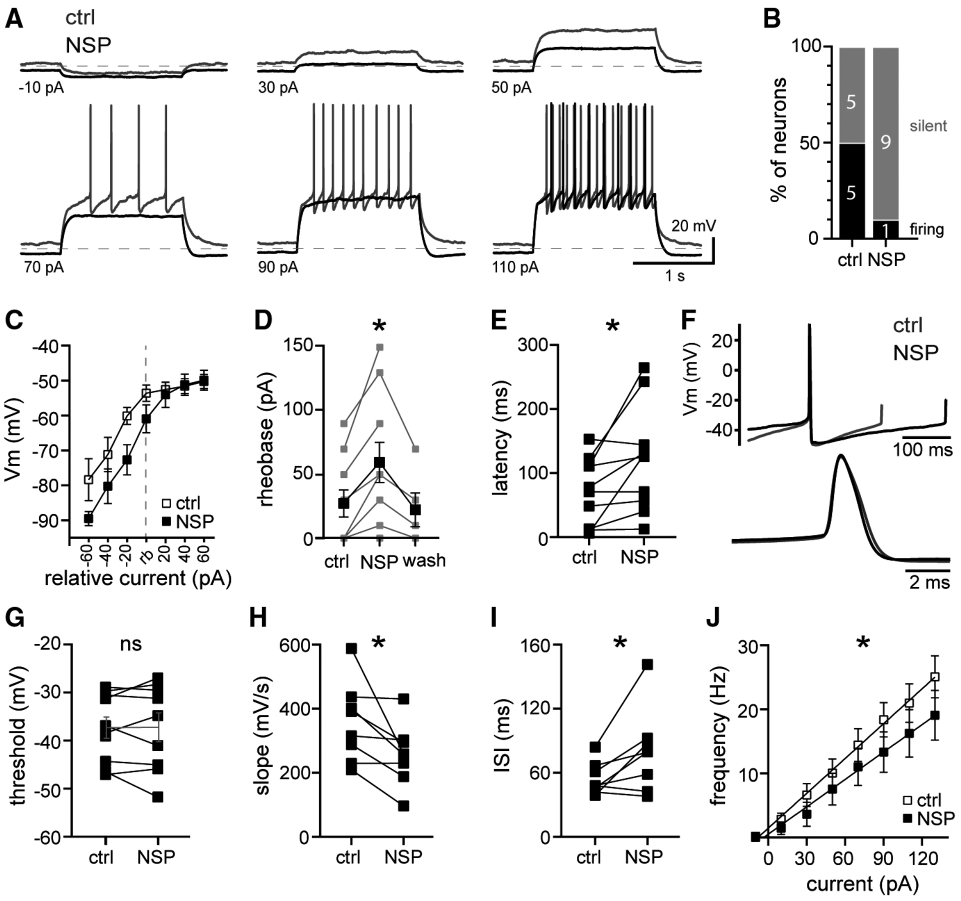

Figure 6. Tonic GluD1$_R$ current provides subthreshold drive of action potential firing.

- A Representative traces of whole-cell current clamp recordings of membrane potential and AP firing evoked by current injection (1.5 s) demonstrating hyperpolarization by NASPM. Dashed line is at −80 mV.
- B Distribution of firing response in control (ctrl) and after application of NASPM (NSP). In control conditions 5/10 neurons fired spontaneously without current injection and 5/10 were silent. In the same neurons after NASPM, 1/10 fired spontaneously and 9/10 were silent.
- C Plot of the membrane potential (Vm) versus injected current relative to rheobase (rb) in control and NASPM, demonstrating that NASPM produced a hyperpolarization at subthreshold potentials (n = 10 biological replicates).
- D Plot of the minimum current needed to induce firing (approximate rheobase) in control conditions, NASPM, and following wash out of NASPM (10 min; P = 0.005, n = 5–10 biological replicates).
- E NASPM increased the latency to fire the first AP upon current injection (150 pA, P = 0.01, n = 10 biological replicates).
- F Average AP waveform recorded in control and in NASPM, aligned at peaks. Below, expanded timescale.
- G NASPM had no effect on the AP threshold (150 pA, measured from the 2nd AP, P < 0.99, n = 9 biological replicates).
- H NASPM decreased the slope of the voltage trajectory between APs (90 pA, measured in the middle 60% of the interspike interval of the first five APs, P = 0.02, n = 8 biological replicates).
- I NASPM increased the interspike interval during evoked firing (90 pA, averaged from first five APs, P = 0.04, n = 8 biological replicates).
- J Plot of the initial firing frequency (first three APs) versus injected current in control and NASPM (P = 0.01, n = 10 biological replicates).

Data information: In (C–E and G–J), line and error bars represent mean ± SEM. ns denotes not significant (D: one-way mixed effects ANOVA, E and G–I: Wilcoxon matched-pairs signed rank tests; J: two-way ANOVA).
Source data are available online for this figure.

(P = 0.19), after-hyperpolarization (P = 0.38), height (P = 0.06), or threshold (Fig 6G). But there was a significant decrease in the slope of the voltage trajectory between APs (Fig 6F and H), resulting in a delay to the next AP (interspike interval, Fig 6I). Overall, NASPM reduced AP firing frequency (measured from the first three APs, Fig 6J). Thus, at physiological levels of extracellular calcium, tonic GluD1$_R$ current contributes to subthreshold drive of action potential firing.

Discussion

Ion channel function of GluD1 receptors

GluD1$_R$ and GluD2$_R$ carry ionic current following the activation of either mGluR or α1-A$_R$ (Ady *et al*, 2013; Dadak *et al*, 2017; Benamer *et al*, 2018; Gantz *et al*, 2020; Lemoine *et al*, 2020). GluD2$_R$ current, seen upon mGluR1 activation in cell lines, is dependent on

© 2023 The Authors

canonical GqPCR signaling as the agonist-induced current is blocked by bath application of $G\alpha_q$ or phospholipase C inhibitors (Dadak *et al*, 2017). Similarly, $GluD1_R$ ionic current activated by $\alpha 1$-A_R in dorsal raphe neurons is abolished after internal dialysis with GDPβS-Li_3 (Gantz *et al*, 2020), a nonspecific disruptor of G protein activity and other processes requiring a GDP-GTP exchange, demonstrating a cell-autonomous requirement of intact G protein signaling. Following our original report of tonic $GluD1_R$ current in brain slices (Gantz *et al*, 2020), Lemoine *et al* (2020) reported a similar tonic current carried by $GluD2_R$ when expressed in cell lines with mGluR1. mGluR, like many GPCRs, can exhibit constitutive activity in the absence of agonist (Prézeau *et al*, 1996), and low-level constitutive activity of GPCRs affects other subthreshold cation conductances (Lu *et al*, 2010; Shen *et al*, 2012; Zhang *et al*, 2012; Quallo *et al*, 2017; Philippart & Khaliq, 2018). Here we show that tonic $GluD1_R$ current is not produced by low-level, cell-autonomous activation of GPCRs. Augmentation of ongoing G protein activity amplified tonic potassium current carried by GIRK channels, but not tonic $GluD1_R$ current. Further, depletion of cell-autonomous G protein activity did not change the amplitude of the tonic $GluD1_R$ current. Thus, tonic $GluD1_R$ current arises from a mechanism separate from ongoing GPCR activity.

In addition to modulation by GPCRs, $GluD1_R$ and $GluD2_R$ are regulated by external glycine and D-serine. These amino acids are known to inhibit constitutively active mutant $GluD1_R$ and $GluD2_R$ "*Lurcher*" channels (Naur *et al*, 2007; Yadav *et al*, 2011) and GPCR-stimulated $GluD1_R$ and $GluD2_R$ currents (Ady *et al*, 2013; Benamer *et al*, 2018; Gantz *et al*, 2020). However, D-serine converts from an inhibitor to an agonist of $GluD2_R$ *Lurcher* channels, when the dimer interface is stabilized while reducing conformational constraints in the ligand-binding domain (Hansen *et al*, 2009). Glycine and D-serine can also open wild-type $GluD2_R$ in HEK-293T cell clusters or synaptically coupled cultured cerebellar neurons through a gating mechanism that requires binding of the N-terminal domains to presynaptic scaffold proteins or otherwise constraining movement in the N-terminal domains with cysteine-cross-linking (Carrillo *et al*, 2021). In contrast, we find that $GluD1_R$ current was unaffected by millimolar glycine or D-serine. Neither amino acid produced a significant inward current nor affected the magnitude of the $\alpha 1$-A_R-stimulated $GluD1_R$ current or tonic $GluD1_R$ current. Therefore, it may be that glycine and D-serine do not directly gate $GluD1_R$, as observed for $GluD2_R$ (Carrillo *et al*, 2021); yet another unidentified endogenous ligand for $GluD1_R$ cannot be ruled out. Alternatively, tonic $GluD1_R$ current may be a product of a low conductance state of $GluD1_R$, akin to "steady-state" current produced by conducting desensitized $GluA_R$ (Coombs *et al*, 2019). If tonic $GluD1_R$ current is a product of desensitized low-conductance $GluD1_R$, then conditions that prevent desensitization (e.g., increasing extracellular calcium, Hansen *et al*, 2009) may paradoxically reduce $GluD1_R$ current. Indeed, we found that $\alpha 1$-A_R-stimulated and tonic $GluD1_R$ currents were bidirectionally regulated by extracellular calcium: increasing extracellular calcium reduced $GluD1_R$ current and decreasing extracellular calcium augmented $GluD1_R$ current. In addition, increasing extracellular calcium slowed the decay rate of the $\alpha 1$-A_R-stimulated $GluD1_R$ current, which may reflect slowing of channel desensitization. High levels of extracellular calcium also "protected" $GluD1_R$ from open-channel block by NASPM. Thus, extracellular calcium has direct inhibitory action on native $GluD1_R$ current in brain slices, either by reducing open channel probability or directly blocking the channel. Future work is needed to distinguish between these possibilities, and it is important to note that several mechanisms may be at play since extracellular calcium is known to affect $GluN_R$ gating through multiple binding sites (Maki & Popescu, 2014).

Importance of tonic cation conductances in excitability

Throughout the central nervous system, many types of neurons fire action potentials in a rhythmic "pacemaker" pattern. Some are autonomous pacemakers, driven by intrinsic membrane properties, while others are conditional pacemakers that rely on synaptic input and receptor stimulation. A common feature in autonomous pacemakers is the presence of a tonic, subthreshold, tetrodotoxin-insensitive, cation/sodium current (Raman *et al*, 2000; Jackson *et al*, 2004; Lu *et al*, 2007; Khaliq & Bean, 2010; Eggermann *et al*, 2011; Li *et al*, 2021). While many different types of channels are involved, these tonic currents each function to depolarize the membrane to ~−60 mV where voltage-dependent mechanisms of action potential firing are engaged. Primarily, serotonin neurons are conditional pacemakers and require subthreshold drive from noradrenergic afferents and activation of $\alpha 1$-A_R (Baraban *et al*, 1978; Vandermaelen & Aghajanian, 1983) much like other conditional pacemakers, which require activation of $G\alpha_q$-coupled orexin or muscarine receptors (Egorov *et al*, 2002, 2019; van den Top *et al*, 2004; Yamada-Hanff & Bean, 2013). In these neurons, activation of GqPCRs leads to subthreshold (~−70 to −55 mV) depolarization via a very similar cation current as the tonic current observed in autonomous pacemakers. While these tonic cation currents are essential for subthreshold depolarization, it is not unusual for the current to be quite small, only a few to tens of picoamperes (Raman *et al*, 2000; Taddese & Bean, 2002; Jackson *et al*, 2004).

Here we show that $GluD1_R$ carried tonic cation current of ~−17 pA at subthreshold potentials (−80 to −55 mV), which depolarized the membrane by ~7 mV. Block of tonic $GluD1_R$ current silenced a subset of dorsal raphe neurons that were firing spontaneously in the brain slice. Under conditions of $GluD1_R$ channel block, dorsal raphe neurons required more somatic current injection to fire. Block of tonic $GluD1_R$ current had little effect on the shape of the APs but prolonged the interval between APs, consistent with a reversal potential of ~0 mV and intrinsic inward rectification (Gantz *et al*, 2020). However, it should be noted that NASPM preferentially blocks inward flow, and strong depolarization relieves pore block (Koike *et al*, 1997). Thus, observation of any contribution of outward ion flux may be obscured. It is worth noting that tonic or steady-state current is not an unusual feature of $GluD1_R$. Long-lasting synaptic currents and tonic currents are observed by the other members of the iGluR family: $GluN_R$ (Sah *et al*, 1989; Misra *et al*, 2000; Meur *et al*, 2007; Chiu & Jahr, 2017; Hanson *et al*, 2019), $kainate_R$ (Castillo *et al*, 1997), and $GluA_R$ either when recovering from desensitization in continued presence of glutamate (Lu *et al*, 2017) or when conducting while desensitized (Coombs *et al*, 2019). Further, desensitization-resistant $GluA_R$ carries a long-lasting "pedestal" current in CA1 pyramidal neurons, which powerfully influences whether a fast synaptic transmission event triggers an action potential (Pampaloni *et al*, 2021). Provided the widespread distribution in the brain (Konno *et al*, 2014; Hepp *et al*, 2015; Nakamoto *et al*, 2020), $GluD1_R$ may contribute to pacemaking in

© 2023 The Authors

other neuronal populations, whether via intrinsic tonic current or following GqPCR activation.

Conclusions

Our results show that $GluD1_R$ carries a G protein-independent tonic current that contributes to subthreshold neuronal excitation in the dorsal raphe nucleus. While the cause of tonic $GluD1_R$ current remains to be resolved, we identified an important part of our recording conditions that support measurement of this current—maintaining extracellular calcium at a physiological level (1.2 mM, Forsberg *et al*, 2019). Increasing extracellular calcium above 2 mM, which is standard to many artificial cerebral spinal fluids, reduced $GluD1_R$ unitary current and the magnitude of tonic $GluD1_R$ by ~50%.

Many studies have demonstrated the "non-ionic" functions of $GluD1_R$ in synapse formation and composition that require binding to presynaptic neurexins and secreted cerebellins (Tao *et al*, 2018; Dai *et al*, 2021). Work by Lemoine *et al* (2020) using HEK-293T cells expressing $GluD2_R$ indicates that stabilization of $GluD2_R$ in a trans-synaptic complex with presynaptic neurexins and secreted cerebellins is not required strictly for GPCR-stimulated and tonic $GluD2_R$ current, in contrast to recent observations with glycine-gated $GluD2_R$ current (Carrillo *et al*, 2021). But interestingly, genetic deletion of cerebellin-2 from dorsal raphe produces hyperactivity, hyperaggression, and compulsive behaviors in mice (Seigneur *et al*, 2021) that are similar to behaviors observed after global deletion of $GluD1_R$ (Yadav *et al*, 2012; Gupta *et al*, 2015). Further, genetic deletion of cerebellin-2 from dorsal raphe reduces serotonin levels in projection areas (Seigneur *et al*, 2021). However, future work will be needed to determine whether $GluD1_R$ ion channel function is potentiated by mechanical stabilization in the trans-synaptic complex or with other accessory proteins.

Materials and Methods

Animals

All studies were conducted in accordance with the University of Iowa with the approval of the University of Iowa Institutional Animal Care and Use Committee. Male and female wild-type C57BL/6J (> 2 months old, The Jackson Laboratory, #000664) mice were used. Mice were group-housed on a 12:12 h light cycle.

Brain slice preparation and electrophysiological recordings

Brain slices and electrophysiological recordings were made as previously described (Khamma *et al*, 2022). In brief, mice were deeply anesthetized with isoflurane and euthanized by decapitation. Brains were removed and placed in warmed and bubbled (95/5% O_2/CO_2) modified Krebs' buffer containing (in mM): 126 NaCl, 2.5 KCl, 1.2 $MgCl_2$, 1.2 $CaCl_2$, 1.2 NaH_2PO_4, 21.5 $NaHCO_3$, and 11 D-glucose with 5 μM MK-801 to reduce excitotoxicity and increase slice viability. In the same solution, coronal dorsal raphe slices (240 μm) were obtained using a vibrating microtome (Leica VT1000S) and incubated at 28°C > 30 min prior to recording.

Electrophysiological recordings were made in modified Krebs' buffer containing NBQX (3 μM) at 35°C with Multiclamp 200B and 700B amplifiers (Molecular Devices), Digidata 1440A and 1550B A/D converters (Molecular Devices), and Clampex software (Molecular Devices) with borosilicate glass electrodes (World Precision Instruments) wrapped with Parafilm to reduce pipette capacitance. Pipette resistances were 3.8–4.5 MΩ when filled with an internal solution containing, (in mM) 104.56 K-methylsulfate, 3.73 KCl, 5.3 NaCl, 4.06 $MgCl_2$, 4.06 $CaCl_2$, 7.07 HEPES (K), 3.25 BAPTA (K4), 0.26 GTP (sodium salt), 4.87 ATP (sodium salt), 4.59 creatine phosphate (sodium salt), pH 7.24 with KOH, mOsm ~274, for whole-cell patch-clamp recordings. Current–voltage relationships of tonic $GluD1_R$ current were determined using voltage ramps from −120 to 10 mV (1 mV/10 ms) including a voltage-gated sodium channel blocker, QX-314 (2 mM) in the internal solution. Current–voltage relationships of GIRK currents were determined using voltage ramps from −50 mV to −130 mV (−0.8 mV/ms). Synaptic currents were evoked on 90-s intervals by applying brief pulses (0.5 ms, 60 Hz) of electrical stimulation to the brain slice via a borosilicate glass monopolar stimulating electrode (World Precision Instruments) placed within 200 μm of the recorded neuron in the presence of GluN (MK-801), GluA/GluK (NBQX, 3 μM), $GABA_A$ (picrotoxin, 100 μM), and 5-HT1a (WAY-100635, 300 nM) receptor blockers to isolate the α1-A_R-EPSC. Series resistance was monitored throughout the experiment. Reported voltages are corrected for a liquid junction potential of −8 mV between the internal and external solution. All drugs were applied via the patch pipette or by bath application. Noradrenaline was applied in the presence of an α2-adrenergic antagonist, idazoxan (1 μM).

Materials

MK-801, NASPM, NBQX, noradrenaline, and prazosin were obtained from Tocris. All other reagents were from Sigma-Aldrich.

Experimental design and statistical analysis

Data were analyzed using Clampfit 10.7 and 11.1 (Molecular Devices) or Igor-Pro 6.37 (Wavemetrics) with DataAccess (Bruxton Corporation) software and are presented in representative traces, scatter plots, and bar graphs with means ± SEM. Unless otherwise noted, n = number of cells as biological replicates. The investigators were not blind to the experimental conditions. To estimate sample size, we conducted power analyses ($\alpha = 0.05$, $\beta = 0.2$) based on detecting a 30% change in α1-A_R-EPSC parameters or a 20 pA change in whole-cell current using standard deviations from published data with similar effect sizes (Gantz *et al*, 2020). Tonic current was measured as the peak of the NASPM-induced current minus the whole-cell current prior to NASPM application. Reversal potentials were determined using linear fit of the averaged data accounting for scatter among the replicates using at least one data point above and two below where the current reversed polarity. Significant differences were determined via Wilcoxon matched-pairs signed rank or two-way ANOVA tests for within-group comparisons and Mann–Whitney or Kruskal-Wallis tests for between-group comparisons. A difference of $P < 0.05$ was considered significant. Exact values are reported unless $P < 0.0001$ or > 0.999. Statistical analysis was performed using GraphPad Prism (GraphPad Software, Inc.).

© 2023 The Authors

Data availability

Data have not been deposited in public databases, but are available upon request.

Expanded View for this article is available online.

Acknowledgements

We thank Holly S. Hake for critical reading and comments on the manuscript. This research was funded by awards from the Roy J. and Lucille A. Carver College of Medicine, University of Iowa, and Roy J. Carver Charitable Trust to SCG.

Author contributions

Daniel S Copeland: Data curation; formal analysis; investigation; visualization; writing – original draft; writing – review and editing. **Aleigha Gugel:** Data curation; formal analysis; investigation; writing – original draft; writing – review and editing. **Stephanie C Gantz:** Conceptualization; data curation; formal analysis; supervision; funding acquisition; investigation; visualization; methodology; writing – original draft; writing – review and editing.

Disclosure and competing interests statement

The authors declare that they have no conflict of interest.

References

Ady V, Perroy J, Tricoire L, Piochon C, Dadak S, Chen X, Dusart I, Fagni L, Lambolez B, Levenes C (2013) Type 1 metabotropic glutamate receptors (mGlu1) trigger the gating of GluD2 delta glutamate receptors. *EMBO Rep* 15: 103 – 109

Araki K, Meguro H, Kushiya E, Takayama C, Inoue Y, Mishina M (1993) Selective expression of the glutamate receptor channel delta 2 subunit in cerebellar Purkinje cells. *Biochem Biophys Res Commun* 197: 1267 – 1276

Baraban JM, Wang RY, Aghajanian G (1978) Reserpine suppression of dorsal raphe neuronal firing: mediation by adrenergic system. *Eur J Pharmacol* 52: 27 – 36

Benamer N, Marti F, Luján R, Hepp R, Aubier TG, Dupin AAM, Frébourg G, Pons S, Maskos U, Faure P *et al* (2018) GluD1, linked to schizophrenia, controls the burst firing of dopamine neurons. *Mol Psychiatry* 23: 691 – 700

Bond RA, IJzerman AP (2006) Recent developments in constitutive receptor activity and inverse agonism, and their potential for GPCR drug discovery. *Trends Pharmacol Sci* 27: 92 – 96

Brown RE, Sergeeva OA, Eriksson KS, Haas HL (2002) Convergent excitation of dorsal raphe serotonin neurons by multiple arousal systems (orexin/hypocretin, histamine and noradrenaline). *J Neurosci* 22: 8850 – 8859

Carrillo E, Gonzalez CU, Berka V, Jayaraman V (2021) Delta glutamate receptors are functional glycine- and D-serine–gated cation channels in situ. *Sci Adv* 7: eabk2200

Castillo PE, Malenka RC, Nicoll RA (1997) Kainate receptors mediate a slow postsynaptic current in hippocampal CA3 neurons. *Nature* 388: 182 – 186

Chiu DN, Jahr CE (2017) Extracellular glutamate in the nucleus accumbens is nanomolar in both synaptic and non-synaptic compartments. *Cell Rep* 18: 2576 – 2583

Chua HC, Wulf M, Weidling C, Rasmussen LP, Pless SA (2020) The NALCN channel complex is voltage sensitive and directly modulated by extracellular calcium. *Sci Adv* 6: eaaz3154

Coombs ID, Soto D, McGee TP, Gold MG, Farrant M, Cull-Candy SG (2019) Homomeric GluA2(R) AMPA receptors can conduct when desensitized. *Nat Commun* 10: 4312

Dadak S, Bouquier N, Goyet E, Fagni L, Levenes C, Perroy J (2017) mGlu1 receptor canonical signaling pathway contributes to the opening of the orphan GluD2 receptor. *Neuropharmacology* 115: 92 – 99

Dai J, Patzke C, Liakath-Ali K, Seigneur E, Südhof TC (2021) GluD1 is a signal transduction device disguised as an ionotropic receptor. *Nature* 595: 1 – 21

Eggermann E, Bucurenciu I, Goswami SP, Jonas P (2011) Nanodomain coupling between Ca2+ channels and sensors of exocytosis at fast mammalian synapses. *Nat Rev Neurosci* 13: 7 – 21

Egorov AV, Hamam BN, Fransén E, Hasselmo ME, Alonso AA (2002) Graded persistent activity in entorhinal cortex neurons. *Nature* 420: 173 – 178

Egorov AV, Schumacher D, Medert R, Birnbaumer L, Freichel M, Draguhn A (2019) TRPC channels are not required for graded persistent activity in entorhinal cortex neurons. *Hippocampus* 29: 1038 – 1048

Forsberg M, Seth H, Björefeldt A, Lyckenvik T, Andersson M, Wasling P, Zetterberg H, Hanse E (2019) Ionized calcium in human cerebrospinal fluid and its influence on intrinsic and synaptic excitability of hippocampal pyramidal neurons in the rat. *J Neurochem* 149: 452 – 470

Fossati M, Assendorp N, Gemin O, Colasse S, Dingli F, Arras G, Loew D, Charrier C (2019) Trans-synaptic signaling through the glutamate Receptor Delta-1 mediates inhibitory synapse formation in cortical pyramidal neurons. *Neuron* 104: 1081 – 1094

Gantz SC, Bunzow JR, Williams JT (2013) Spontaneous inhibitory synaptic currents mediated by a G protein-coupled receptor. *Neuron* 78: 807 – 812

Gantz SC, Moussawi K, Hake HS (2020) Delta glutamate receptor conductance drives excitation of mouse dorsal raphe neurons. *Elife* 9: e56054

Gawande DY, Shelkar GP, Liu J, Ayala AD, Pavuluri R, Choi D, Smith Y, Dravid SM (2021) Glutamate Delta-1 receptor regulates inhibitory neurotransmission in the nucleus Accumbens Core and anxiety-like behaviors. *Mol Neurobiol* 58: 4787 – 4801

Gawande DY, Narasimhan KKS, Bhatt JM, Pavuluri R, Kesherwani V, Suryavanshi PS, Shelkar GP, Dravid SM (2022) Glutamate delta 1 receptor regulates autophagy mechanisms and affects excitatory synapse maturation in the somatosensory cortex. *Pharmacol Res* 178: 106144

Gupta SC, Yadav R, Pavuluri R, Morley BJ, Stairs DJ, Dravid SM (2015) Essential role of GluD1 in dendritic spine development and GluN2B to GluN2A NMDAR subunit switch in the cortex and hippocampus reveals ability of GluN2B inhibition in correcting hyperconnectivity. *Neuropharmacology* 93: 274 – 284

Hansen KB, Naur P, Kurtkaya NL, Kristensen AS, Gajhede M, Kastrup JS, Traynelis SF (2009) Modulation of the dimer interface at ionotropic glutamate-like receptor 2 by D-serine and extracellular calcium. *J Neurosci* 29: 907 – 917

Hanson E, Armbruster M, Lau LA, Sommer ME, Klaft Z-J, Swanger SA, Traynelis SF, Moss SJ, Noubary F, Chadchankar J *et al* (2019) Tonic activation of GluN2C/GluN2D-containing NMDA receptors by ambient glutamate facilitates cortical interneuron maturation. *J Neurosci* 39: 3611 – 3626

Hein P, Goepel M, Cotecchia S, Michel MC (2001) A quantitative analysis of antagonism and inverse agonism at wild-type and constitutively active hamster α1B-adrenoceptors. *Naunyn Schmiedebergs Arch Pharmacol* 363: 34 – 39

Hepp R, Hay YA, Aguado C, Luján R, Dauphinot L, Potier MC, Nomura S, Poirel O, Mestikawy SE, Lambolez B *et al* (2015) Glutamate receptors of the delta family are widely expressed in the adult brain. *Brain Struct Funct* 220: 2797 – 2815

 © 2023 The Authors

Hoover AH, Pavuluri R, Shelkar GP, Dravid SM, Smith Y, Villalba RM (2021) Ultrastructural localization of glutamate delta 1 (GluD1) receptor immunoreactivity in the mouse and monkey striatum. *J Comp Neurol* 529: 1703 – 1718

Hubbard CM, Redpath GT, Macdonald TL, VandenBerg SR (1989) Modulatory effects of aluminum, calcium, lithium, magnesium, and zinc ions on [3H]MK-801 binding in human cerebral cortex. *Brain Res* 486: 170 – 174

Jackson AC, Yao GL, Bean BP (2004) Mechanism of spontaneous firing in dorsomedial suprachiasmatic nucleus neurons. *J Neurosci* 24: 7985 – 7998

Khaliq ZM, Bean BP (2010) Pacemaking in dopaminergic ventral tegmental area neurons: depolarizing drive from background and voltage-dependent sodium conductances. *J Neurosci* 30: 7401 – 7413

Khamma JK, Copeland DS, Hake HS, Gantz SC (2022) Spatiotemporal control of noradrenaline-dependent synaptic transmission in mouse dorsal raphe serotonin neurons. *J Neurosci* 42: 968 – 979

Koike M, Iino M, Ozawa S (1997) Blocking effect of 1-naphthyl acetyl spermine on Ca(2+)-permeable AMPA receptors in cultured rat hippocampal neurons. *Neurosci Res* 29: 27 – 36

Konno K, Matsuda K, Nakamoto C, Uchigashima M, Miyazaki T, Yamasaki M, Sakimura K, Yuzaki M, Watanabe M (2014) Enriched expression of GluD1 in higher brain regions and its involvement in parallel fiber-interneuron synapse formation in the cerebellum. *J Neurosci* 34: 7412 – 7424

Kramer PF, Williams JT (2016) Calcium release from stores inhibits GIRK. *Cell Rep* 17: 3246 – 3255

Landsend AS, Amiry-Moghaddam M, Matsubara A, Bergersen L, Usami S, Wenthold RJ, Ottersen OP (1997) Differential localization of δ glutamate receptors in the rat cerebellum: coexpression with AMPA receptors in parallel fiber–spine synapses and absence from climbing fiber–spine synapses. *J Neurosci* 17: 834 – 842

Lemoine D, Mondoloni S, Tange J, Lambolez B, Faure P, Taly A, Tricoire L, Mourot A (2020) Probing the ionotropic activity of glutamate GluD2 receptor in HEK cells with genetically-engineered photopharmacology. *Elife* 9: e59026

Li K, Abbott SBG, Shi Y, Eggan P, Gonye EC, Bayliss DA (2021) TRPM4 mediates a subthreshold membrane potential oscillation in respiratory chemoreceptor neurons that drives pacemaker firing and breathing. *Cell Rep* 34: 108714

Lomeli H, Sprengel R, Laurie DJ, Kohr G, Herb A, Seeburg PH, Wisden W (1993) The rat delta-1 and delta-2 subunits extend the excitatory amino acid receptor family. *FEBS Lett* 315: 318 – 322

Lopes JP, Cunha RA (2019) What is the extracellular calcium concentration within brain synapses? *J Neurochem* 149: 435 – 437

Loucif AJC, Bonnavion P, Macri B, Golmard J-L, Boni C, Melfort M, Leonard G, Lesch K-P, Adrien J, Jacquin TD (2006) Gender-dependent regulation of G-protein-gated inwardly rectifying potassium current in dorsal raphe neurons in knock-out mice devoid of the 5-hydroxytryptamine transporter. *J Neurobiol* 66: 1475 – 1488

Lu B, Su Y, Das S, Liu J, Xia J, Ren D (2007) The neuronal channel NALCN contributes resting sodium permeability and is required for normal respiratory rhythm. *Cell* 129: 371 – 383

Lu B, Su Y, Das S, Wang H, Wang Y, Liu J, Ren D (2009) Peptide neurotransmitters activate a cation channel complex of NALCN and UNC-80. *Nature* 457: 741 – 744

Lu B, Zhang Q, Wang H, Wang Y, Nakayama M, Ren D (2010) Extracellular calcium controls background current and neuronal excitability via an UNC79-UNC80-NALCN Cation Channel complex. *Neuron* 68: 488 – 499

Lu H-W, Balmer TS, Romero GE, Trussell LO (2017) Slow AMPAR synaptic transmission is determined by Stargazin and glutamate transporters. *Neuron* 96: 73 – 80

Maki BA, Popescu GK (2014) Extracellular Ca2+ ions reduce NMDA receptor conductance and gating. *J Gen Physiol* 144: 379 – 392

McKay S, Bengtson CP, Bading H, Wyllie DJA, Hardingham GE (2013) Recovery of NMDA receptor currents from MK-801 blockade is accelerated by Mg2+ and memantine under conditions of agonist exposure. *Neuropharmacology* 74: 119 – 125

Meur KL, Galante M, Angulo MC, Audinat E (2007) Tonic activation of NMDA receptors by ambient glutamate of non-synaptic origin in the rat hippocampus. *J Physiol* 580: 373 – 383

Misra C, Brickley SG, Wyllie DJA, Cull-Candy SG (2000) Slow deactivation kinetics of NMDA receptors containing NR1 and NR2D subunits in rat cerebellar Purkinje cells. *J Physiol* 525: 299 – 305

Nakamoto C, Konno K, Miyazaki T, Nakatsukasa E, Natsume R, Abe M, Kawamura M, Fukazawa Y, Shigemoto R, Yamasaki M *et al* (2020) Expression mapping, quantification, and complex formation of GluD1 and GluD2 glutamate receptors in adult mouse brain. *J Comp Neurol* 528: 1003 – 1027

Naur P, Hansen KB, Kristensen AS, Dravid SM, Pickering DS, Olsen L, Vestergaard B, Egebjerg J, Gajhede M, Traynelis SF *et al* (2007) Ionotropic glutamate-like receptor delta2 binds D-serine and glycine. *Proc Natl Acad Sci USA* 104: 14116 – 14121

Pampaloni NP, Riva I, Carbone A, Plested AJR (2021) Slow AMPA receptors in hippocampal principal cells. *Cell Rep* 35: 109496

Pfaffinger PJ, Martin JM, Hunter DD, Nathanson NM, Hille B (1985) GTP-binding proteins couple cardiac muscarinic receptors to a K channel. *Nature* 317: 536 – 538

Philippart F, Khaliq ZM (2018) Gi/o protein-coupled receptors in dopamine neurons inhibit the sodium leak channel NALCN. *Elife* 7: e40984

Prézeau L, Gomeza J, Ahern S, Mary S, Galvez T, Bockaert J, Pin JP (1996) Changes in the carboxyl-terminal domain of metabotropic glutamate receptor 1 by alternative splicing generate receptors with differing agonist-independent activity. *Mol Pharmacol* 49: 422 – 429

Quallo T, Alkhatib O, Gentry C, Andersson DA, Bevan S (2017) G protein βγ subunits inhibit TRPM3 ion channels in sensory neurons. *Elife* 6: e26138

Raman IM, Gustafson AE, Padgett D (2000) Ionic currents and spontaneous firing in neurons isolated from the cerebellar nuclei. *J Neurosci* 20: 9004 – 9016

Ren D (2011) Sodium leak channels in neuronal excitability and rhythmic behaviors. *Neuron* 72: 899 – 911

Reynolds IJ, Miller RJ (1988) Multiple sites for the regulation of the N-methyl-D-aspartate receptor. *Mol Pharmacol* 33: 581 – 584

Rodriguez-Contreras D, Condon AF, Buck DC, Asad N, Dore TM, Verbeek DS, Tijssen MAJ, Shinde U, Williams JT, Neve KA (2021) Signaling-biased and constitutively active dopamine D2 receptor variant. *ACS Chem Nerosci* 12: 1873 – 1884

Sah P, Hestrin S, Nicoll RA (1989) Tonic activation of NMDA receptors by ambient glutamate enhances excitability of neurons. *Science* 246: 815 – 818

Seigneur E, Wang J, Dai J, Polepalli J, Südhof TC (2021) Cerebellin-2 regulates a serotonergic dorsal raphe circuit that controls compulsive behaviors. *Mol Psychiatry* 26: 7509 – 7521

Shen Y, Rampino MAF, Carroll RC, Nawy S (2012) G-protein-mediated inhibition of the Trp channel TRPM1 requires the G dimer. *Proc Natl Acad Sci USA* 109: 8752 – 8757

Svensson TH, Bunney BS, Aghajanian GK (1975) Inhibition of both noradrenergic and serotonergic neurons in brain by the alpha-adrenergic agonist clonidine. *Brain Res* 92: 291 – 306

© 2023 The Authors

Taddese A, Bean BP (2002) Subthreshold sodium current from rapidly inactivating sodium channels drives spontaneous firing of Tuberomammillary neurons. *Neuron* 33: 587 – 600

Tao W, Díaz-Alonso J, Sheng N, Nicoll RA (2018) Postsynaptic δ1 glutamate receptor assembles and maintains hippocampal synapses via Cbln2 and neurexin. *Proc Natl Acad Sci USA* 115: E5373 – E5381

van den Top M, Lee K, Whyment AD, Blanks AM, Spanswick D (2004) Orexigen-sensitive NPY/AgRP pacemaker neurons in the hypothalamic arcuate nucleus. *Nat Neurosci* 7: 493 – 494

Vandermaelen CP, Aghajanian GK (1983) Electrophysiological and pharmacological characterization of serotonergic dorsal raphe neurons recorded extracellularly and intracellularly in rat brain slices. *Brain Res* 289: 109 – 119

Yadav R, Rimerman R, Scofield MA, Dravid SM (2011) Mutations in the transmembrane domain M3 generate spontaneously open orphan glutamate δ1 receptor. *Brain Res* 1382: 1 – 8

Yadav R, Gupta SC, Hillman BG, Bhatt JM, Stairs DJ, Dravid SM (2012) Deletion of glutamate delta-1 receptor in mouse leads to aberrant emotional and social behaviors. *PLoS ONE* 7: e32969

Yamada-Hanff J, Bean BP (2013) Persistent sodium current drives conditional pacemaking in CA1 pyramidal neurons under muscarinic stimulation. *J Neurosci* 33: 15011 – 15021

Zhang X, Mak S, Li L, Parra A, Denlinger B, Belmonte C, McNaughton PA (2012) Direct inhibition of the cold-activated TRPM8 ion channel by Gαq. *Nat Cell Biol* 14: 851 – 858

License: This is an open access article under the terms of the Creative Commons Attribution License, which permits use, distribution and reproduction in any medium, provided the original work is properly cited.

 © 2023 The Authors

Expanded View Figures

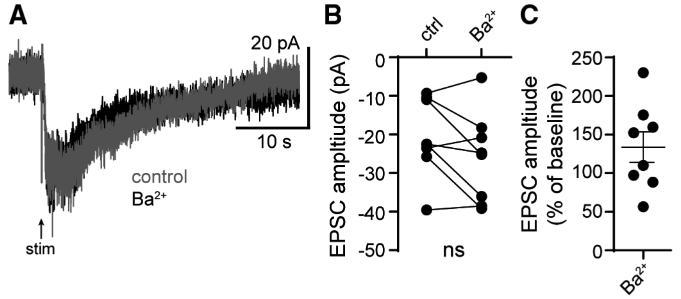

Figure EV1. External Ba^{2+} has no effect on the α1-adrenergic receptor-dependent excitatory p-ostsynaptic current.

A Representative traces of electrically evoked (arrow) α1-A_R-EPSCs in control conditions and after application of Ba^{2+} (100 μM).

B Plot of the amplitude of the α1-A_R-EPSC in control conditions (ctrl) and after application of Ba^{2+} ($P = 0.20$, $n = 8$ biological replicates).

C Plot of the percent change in α1-A_R-EPSC amplitude with external Ba^{2+} ($n = 8$ biological replicates).

Data information: In (B, C), line and error bars represent mean ± SEM. ns denotes not significant (Wilcoxon matched-pairs signed rank test).
Source data are available online for this figure.

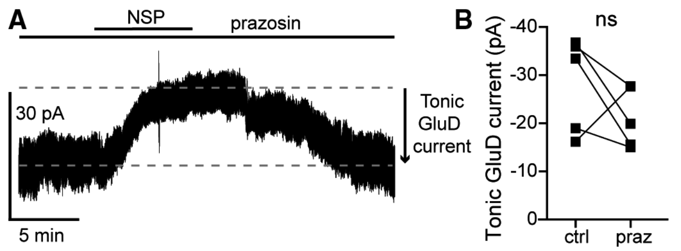

Figure EV2. Inverse agonism of α1-adrenergic receptors has no effect on tonic $GluD1_R$ current.

A Representative whole-cell voltage-clamp recording of the apparent outward current produced by application of NASPM (NSP) in the presence of an α1-adrenergic receptor inverse agonist, prazosin (100 nM).

B Plot of the magnitude of $GluD1_R$ tonic current in control conditions (ctrl) and after application of prazosin (praz; $P = 0.31$, $n = 5$ biological replicates).

Data information: In (B), ns denotes not significant (Wilcoxon matched-pairs signed rank test).
Source data are available online for this figure.

© 2023 The Authors

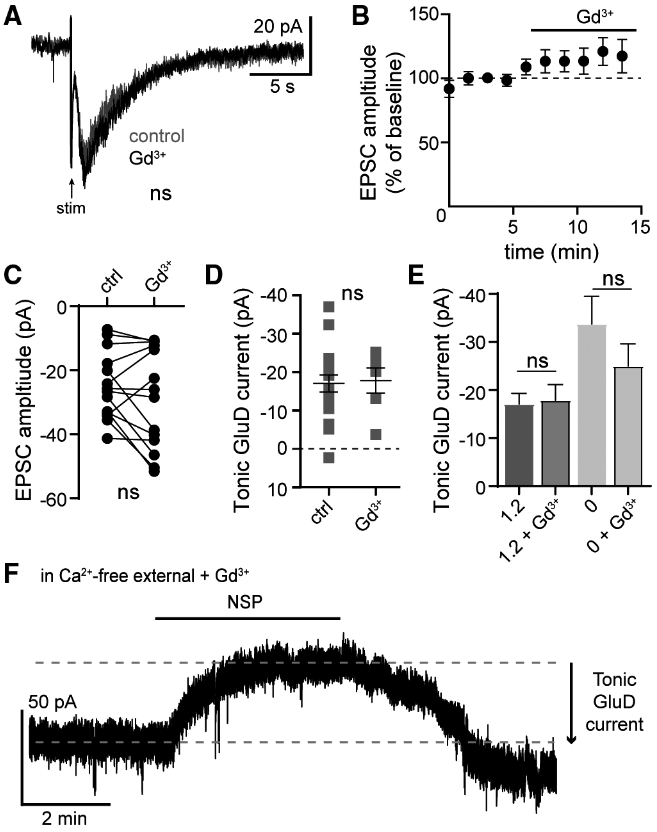

Figure EV3. GluD1$_R$ current is insensitive to gadolinium.

A Representative traces of electrically evoked (arrow) α1-A$_R$-EPSCs in control conditions and after application of Gd^{3+} (10 μM).

B Gd^{3+} had no significant effect on the amplitude of the α1-A$_R$-EPSC, shown in a time-course plot. Dashed line indicates 100% of baseline amplitude (n = 14 biological replicates).

C Plot of the amplitude of the α1-A$_R$-EPSC in control conditions (ctrl) and in Gd^{3+} (10 μM, P = 0.15, n = 14).

D Plot of the magnitude of tonic GluD1$_R$ current measured in control conditions (ctrl), or in the presence of Gd^{3+}, showing no difference in the amplitude of tonic GluD1$_R$ current (P = 0.58, n = 18 and 6 biological replicates).

E Plot of the magnitude of tonic GluD1$_R$ current measured in standard external calcium (1.2 mM) with and without Gd^{3+} (as shown in D) and in calcium-free external solution (0 mM) with and without Gd^{3+}. Gd^{3+} had no significant effect on the magnitude of the tonic GluD1$_R$ current (1.2 vs. 1.2+Gd^{3+}: $P > 0.999$; 1.2 vs. 0: P = 0.024; 1.2+Gd^{3+} vs. 0+Gd^{3+}: $P > 0.99$; 0 vs. 0+Gd^{3+}: $P > 0.99$, n = 18, 18, 6, and 14 biological replicates respectively).

F In nominally calcium-free external and Gd^{3+}, NASPM still produced a large apparent outward current, shown in a representative whole-cell voltage-clamp recording.

Data information: In (B–E) line and error bars represent mean ± SEM, ns denotes not significant (C: Wilcoxon test; D: Mann–Whitney test; E: Kruskal–Wallis test).
Source data are available online for this figure.

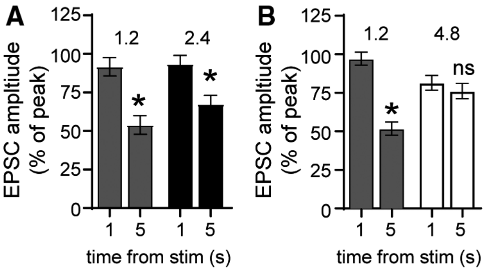

Figure EV4. High extracellular calcium prolongs the α1-adrenergic receptor-dependent excitatory postsynaptic current.

A Plot of the amplitude of the α1-A$_R$-EPSC relative to the peak amplitude, measured 1 and 5 s from stimulation in control conditions (1.2 mM calcium) and after application of 2.4 mM calcium. In both 1.2 and 2.4 mM calcium, the amplitude of the α1-A$_R$-EPSC is reduced significantly by 5 s after stimulation (1.2 mM, P = 0.001; 2.4 mM, P = 0.017, n = 14 biological replicates).

B Plot of the amplitude of the α1-A$_R$-EPSC relative to the peak amplitude, measured 1 and 5 s from stimulation in control conditions (1.2 mM calcium) and after application of 4.8 mM calcium. In 1.2 mM, but not in 4.8 mM calcium, the amplitude of the α1-A$_R$-EPSC is reduced significantly by 5 s after stimulation (1.2 mM, $P < 0.0001$; 4.8 mM, P = 0.671, n = 21 biological replicates).

Data information: Line and error bars represent mean ± SEM. * denotes statistical significance, ns denotes not significant: Two-way ANOVA tests.
Source data are available online for this figure.

 © 2023 The Authors
