# Supplementary material for: Potentiation of neuronal activity by tonic GluD1 current in brain slices
Source: EMBO Rep. 2023 May 8;24(7):e56801. doi: 10.15252/embr.202356801 (PMC10328076; doi:10.15252/embr.202356801)
Supplement: Supplementary file 10 [file EMBR-24-e56801-s003.pdf]

EMBO reports

Potentiation of neuronal activity by tonic GluD1 current in brain slices

Daniel Copeland, Aleigha Gugel, and Stephanie Gantz
DOI: 10.15252/embr.202356801

Corresponding author(s): Stephanie Gantz (Stephanie-Gantz@uiowa.edu)

Review Timeline:

Submission Date:	9th Jan 23
Editorial Decision:	9th Feb 23
Revision Received:	30th Mar 23
Editorial Decision:	17th Apr 23
Revision Received:	19th Apr 23
Accepted:	24th Apr 23

Editor: Esther Schnapp

Transaction Report:

(Note: With the exception of the correction of typographical or spelling errors that could be a source of ambiguity, letters and reports are not edited. Depending on transfer agreements, referee reports obtained elsewhere may or may not be included in this compilation. Referee reports are anonymous unless the Referee chooses to sign their reports.)

Dear Dr. Gantz,

Thank you for the submission of your manuscript to EMBO reports. We have now received the full set of referee reports that is pasted below.

As you will see, the referees are actually quite excited about your study and acknowledge that the findings are interesting. They only have minor comments that should all be addressed.

I would thus like to invite you to revise your manuscript with the understanding that the referee concerns must be fully addressed and their suggestions taken on board. Please address all referee concerns in a complete point-by-point response. Acceptance of the manuscript will depend on a positive outcome of a second round of review. It is EMBO reports policy to allow a single round of major revision only and acceptance or rejection of the manuscript will therefore depend on the completeness of your responses included in the next, final version of the manuscript.

We realize that it is difficult to revise to a specific deadline. In the interest of protecting the conceptual advance provided by the work, we recommend a revision within 3 months (12th May 2023). Please discuss the revision progress ahead of this time with the editor if you require more time to complete the revisions.

You can either publish the study as a short report or as a full article. For short reports, the revised manuscript should not exceed 27,000 characters (including spaces but excluding materials & methods and references) and 5 main plus 5 expanded view figures. The results and discussion sections must further be combined, which will help to shorten the manuscript text by eliminating some redundancy that is inevitable when discussing the same experiments twice. For a normal article there are no length limitations, but it should have more than 5 main figures and the results and discussion sections must be separate. In both cases, the entire materials and methods must be included in the main manuscript file.

IMPORTANT NOTE: we perform an initial quality control of all revised manuscripts before re-review. Your manuscript will FAIL this control and the handling will be DELAYED if the following APPLIES:
1) A data availability section providing access to data deposited in public databases is missing. If you have not deposited any data, please add a sentence to the data availability section that explains that.
2) Your manuscript contains statistics and error bars based on n=2. Please use scatter blots in these cases. No statistics should be calculated if n=2.

When submitting your revised manuscript, please carefully review the instructions that follow below. Failure to include requested items will delay the evaluation of your revision.

1) a .docx formatted version of the manuscript text (including legends for main figures, EV figures and tables). Please make sure that the changes are highlighted to be clearly visible.

2) individual production quality figure files as .eps, .tif, .jpg (one file per figure). See https://wol-prod-cdn.literatumonline.com/pb-assets/embo-site/EMBOPress_Figure_Guidelines_061115-1561436025777.pdf for more info on how to prepare your figures.

3) We replaced Supplementary Information with Expanded View (EV) Figures and Tables that are collapsible/expandable online. A maximum of 5 EV Figures can be typeset. EV Figures should be cited as 'Figure EV1, Figure EV2" etc... in the text and their respective legends should be included in the main text after the legends of regular figures.

- For the figures that you do NOT wish to display as Expanded View figures, they should be bundled together with their legends in a single PDF file called *Appendix*, which should start with a short Table of Content. Appendix figures should be referred to in the main text as: "Appendix Figure S1, Appendix Figure S2" etc. See detailed instructions regarding expanded view here: <https://www.embopress.org/page/journal/14693178/authorguide#expandedview>

- Additional Tables/Datasets should be labeled and referred to as Table EV1, Dataset EV1, etc. Legends have to be provided in a separate tab in case of .xls files. Alternatively, the legend can be supplied as a separate text file (README) and zipped together with the Table/Dataset file.

4) a .docx formatted letter INCLUDING the reviewers' reports and your detailed point-by-point responses to their comments. As part of the EMBO Press transparent editorial process, the point-by-point response is part of the Review Process File (RPF), which will be published alongside your paper.

5) a complete author checklist, which you can download from our author guidelines <https://www.embopress.org/page/journal/14693178/authorguide>. Please insert information in the checklist that is also reflected in the manuscript. The completed author checklist will also be part of the RPF.

6) Please note that all corresponding authors are required to supply an ORCID ID for their name upon submission of a revised manuscript (<https://orcid.org/>). Please find instructions on how to link your ORCID ID to your account in our manuscript tracking system in our Author guidelines
<https://www.embopress.org/page/journal/14693178/authorguide#authorshipguidelines>

7) Before submitting your revision, primary datasets produced in this study need to be deposited in an appropriate public database (see https://www.embopress.org/page/journal/14693178/authorguide#datadeposition). Please remember to provide a reviewer password if the datasets are not yet public. The accession numbers and database should be listed in a formal "Data Availability" section placed after Materials & Method (see also https://www.embopress.org/page/journal/14693178/authorguide#datadeposition). Please note that the Data Availability Section is restricted to new primary data that are part of this study. * Note - All links should resolve to a page where the data can be accessed. *
If your study has not produced novel datasets, please mention this fact in the Data Availability Section.

8) At EMBO Press we ask authors to provide source data for the main manuscript figures. Our source data coordinator will contact you to discuss which figure panels we would need source data for and will also provide you with helpful tips on how to upload and organize the files.

9) Our journal also encourages inclusion of *data citations in the reference list* to directly cite datasets that were re-used and obtained from public databases. Data citations in the article text are distinct from normal bibliographical citations and should directly link to the database records from which the data can be accessed. In the main text, data citations are formatted as follows: "Data ref: Smith et al, 2001" or "Data ref: NCBI Sequence Read Archive PRJNA342805, 2017". In the Reference list, data citations must be labeled with "[DATASET]". A data reference must provide the database name, accession number/identifiers and a resolvable link to the landing page from which the data can be accessed at the end of the reference. Further instructions are available at https://www.embopress.org/page/journal/14693178/authorguide#referencesformat

10) Regarding data quantification (see Figure Legends: https://www.embopress.org/page/journal/14693178/authorguide#figureformat)

The following points must be specified in each figure legend:

- the name of the statistical test used to generate error bars and P values,

- the number (n) of independent experiments (please specify technical or biological replicates) underlying each data point,

- the nature of the bars and error bars (s.d., s.e.m.),

- If the data are obtained from n Program fragment delivered error ``Can't locate object method "less" via package "than" (perhaps you forgot to load "than"?) at //ejpvfs23/sites23b/embor_www/letters/embor_decision_revise_and_review.txt line 56.' 2, use scatter blots showing the individual data points.

Discussion of statistical methodology can be reported in the materials and methods section, but figure legends should contain a basic description of n, P and the test applied.

- Please also include scale bars in all microscopy images.

11) The journal requires a statement specifying whether or not authors have competing interests (defined as all potential or actual interests that could be perceived to influence the presentation or interpretation of an article). In case of competing interests, this must be specified in your disclosure statement. Further information: https://www.embopress.org/competing-interests

We would also welcome the submission of cover suggestions, or motifs to be used by our Graphics Illustrator in designing a cover.

As part of the EMBO publication's Transparent Editorial Process, EMBO reports publishes online a Review Process File (RPF) to accompany accepted manuscripts. This File will be published in conjunction with your paper and will include the referee reports, your point-by-point response and all pertinent correspondence relating to the manuscript.

You are able to opt out of this by letting the editorial office know (emboreports@embo.org). If you do opt out, the Review Process File link will point to the following statement: "No Review Process File is available with this article, as the authors have chosen not to make the review process public in this case."

I look forward to seeing a revised form of your manuscript when it is ready. Please use this link to submit your revision: https://embor.msubmit.net/cgi-bin/main.plex

Best regards,
Esther

Esther Schnapp, PhD
Senior Editor
EMBO reports

Referee #1:

In this manuscript, authors report the novel and important finding that in dorsal raphe neurons, endogenous GluD1 Receptors carry a tonic current that is independent of ongoing G-protein activity and is sensitive to variations in external Ca in the physiologic range; further, they show that this ongoing GluD1 receptor-mediated current modulates the excitability of these neurons.
This is an important finding because although homologous in structure with the principal glutamatergic excitatory channels, encoded by GluA (AMPA), GluK (kainate), and GluN (NMDA) genes, receptors encoded by GluD1 and GluD2 genes remain poorly understood in terms of both gating and modulation and role in neurophysiology. This gap in knowledge has persisted because in recombinant systems, standard pharmacologic approaches have failed to elicit ligand-dependent currents from GluD receptors. Disparate reports suggest that GluD receptor currents may respond to intracellular signaling cascades, such as those mediated by G proteins, or kinase/phosphatases, or mechanically stabilizing structural connections with adjacent cells. Work from this group has provided provocative evidence, reproduced here in figure 1, that GluD1 receptors mediate a small (~20 pA) but tonic excitatory current, which reduces the resting membrane potential of neurons and elevates their excitability.
In this report, the authors provide additional evidence that the tonic current is independent of G protein-signaling and demonstrate for the first time that this current is sensitive to variations in extracellular Ca in the physiologic range. The take home message is exciting and of broad interest to those seeking to elucidate excitatory transmission in the CNS, and opens new vistas of research.
Several laboratories are seeking to elucidate the role of GluD 1 in synaptic transmission and plasticity, and the functionality of GluD receptors as ion channels remains controversial. Therefore, the message is timely and needs to be reported soon.
The experiments are rigorous and pertinent to the questions asked. As a suggestion for improvement, the text can be made more accessible to a broader audience by a thorough editing.

Referee #2:

This is a thorough investigation of the tonic GluD1 current. I find the results convincing. It is sometimes hard to be absolutely sure about leak currents, but the finding that calcium can be used to inhibit is most useful. The work is carefully and thoughtfully done and the paper is nicely written. I found myself learning facts about leak currents that I did not know. Thank you. I have just one major point that refers to writing more clearly.

1. Reading the manuscript with a cynical eye, I was asking myself how do we actually know that the putative GluD1 tonic current is in fact not NALCN. The authors do not mention NALCN explicitly in the text but do cite some references that indicate the NALCN leak currents should be affected by G-protein coupled activity (which is not the case of the tonic current here). I think it would be better to be specific on this exclusion criteria. The tonic current is gone in the case of GluD1 KO, I realise (eLife 2020 paper). But as it the putative GluD1 leak current examined here seems independent of several other GluD1-specific mechanisms, I think it's wise to give as much explicit information as possible, as to why this possibility (NALCN) is excluded.

This is particularly important because in previous work (eLife 2020 paper), the senior author wrote that "a1-adrenergic receptors modulate tonic GluD1R-channel current" even if, in hindsight, this is not really what was meant and isn't the case.

One last point, for future work: In an ideal world the GluD1 tonic current that increases in zero calcium would be controlled against KO cells (as was done before for the 2020 eLife paper). That is, the KO should have no calcium dependent NASPM sensitive leak current.

minor
line 361 All other critical reagents were from Sigma-Aldrich.
Why make the distinction? What are non-critical reagents?

Figure 6 "Tonic GluD1R current is reduced by elevated levels of extracellular calcium."
perhaps it would be better just to say "inhibited by extracellular calcium" ? It's not reduced by elevated calcium in any

physiological context, but it is in fact tonically reduced by the normal level of calcium. Perhaps this is just a matter of taste but I think you can be more direct.

the labelling in figure 5A is misleading - there was external Ca2+ throughout.

Fig 1E : is the x-axis reversed and positive in voltage? Surely not. Can you explain what the downward blip is around -30 mV? Calcium channels?

Fig 2B: same problem as in Fig 1E? Missing minus signs?

Referee #3:

Summary
This manuscript reports the finding that the tonic GluD1R current is modulated, not by G protein signaling, but instead by extracellular Ca concentration in cells in the dorsal raphe nucleus. This work is significant because it shows that these receptors are modulated by Ca and contribute to resting membrane potential and excitability. Any mechanisms that controls neuronal excitability so strongly should be of interest to the molecular biology community. The finding is robustly documented and alternative hypotheses are tested, including the roles of extracellular D-serine and glycine, that have been shown to gate GluD channels in other preparations. Strengths of this work is the use of adult animals of both sexes. The experiments are focused, the analyses are appropriate overall and the writing is well structured and concise.

Minor comments
Fig 1e, 2b- X-axis should be negative
Line 309- Unclear what is meant by the word origin in 'the origin of tonic GluD1R current

Referee #1:

In this manuscript, authors report the novel and important finding that in dorsal raphe neurons, endogenous GluD1 Receptors carry a tonic current that is independent of ongoing G-protein activity and is sensitive to variations in external Ca in the physiologic range; further, they show that this ongoing GluD1 receptor-mediated current modulates the excitability of these neurons.

This is an important finding because although homologous in structure with the principal glutamatergic excitatory channels, encoded by GluA (AMPA), GluK (kainate), and GluN (NMDA) genes, receptors encoded by GluD1 and GluD2 genes remain poorly understood in terms of both gating and modulation and role in neurophysiology. This gap in knowledge has persisted because in recombinant systems, standard pharmacologic approaches have failed to elicit ligand-dependent currents from GluD receptors. Disparate reports suggest that GluD receptor currents may respond to intracellular signaling cascades, such as those mediated by G proteins, or kinase/phosphatases, or mechanically stabilizing structural connections with adjacent cells. Work from this group has provided provocative evidence, reproduced here in figure 1, that GluD1 receptors mediate a small (~20 pA) but tonic excitatory current, which reduces the resting membrane potential of neurons and elevates their excitability.

In this report, the authors provide additional evidence that the tonic current is independent of G protein-signaling and demonstrate for the first time that this current is sensitive to variations in extracellular Ca in the physiologic range. The take home message is exciting and of broad interest to those seeking to elucidate excitatory transmission in the CNS, and opens new vistas of research.

Several laboratories are seeking to elucidate the role of GluD 1 in synaptic transmission and plasticity, and the functionality of GluD receptors as ion channels remains controversial. Therefore, the message is timely and needs to be reported soon.

The experiments are rigorous and pertinent to the questions asked. As a suggestion for improvement, the text can be made more accessible to a broader audience by a thorough editing.

Thank you for your critique. We have edited the manuscript with accessibility in mind.

Referee #2:

This is a thorough investigation of the tonic GluD1 current. I find the results convincing. It is sometimes hard to be absolutely sure about leak currents, but the finding that calcium can be used to inhibit is most useful. The work is carefully and thoughtfully done and the paper is nicely written. I found myself learning facts about leak currents that I did not know. Thank you. I have just one major point that refers to writing more clearly.

1. Reading the manuscript with a cynical eye, I was asking myself how do we actually know that the putative GluD1 tonic current is in fact not NALCN. The authors do not mention NALCN explicitly in the text but do cite some references that indicate the NALCN leak currents should be affected by G-protein coupled activity (which is not the case of the tonic current here). I think it would be better to be specific on this exclusion criteria.

This is an excellent point and we greatly appreciate the opportunity to address possible involvement of NALCN directly. We have edited the text to explicitly clarify how NALCN is affected by G protein coupled activity and how it is different from what we observe for GluD1.

NALCN current is stimulated by GqPCR activation in a G protein-independent manner (Lu *et al*, 2009). There is also a 'tonic' NALCN current that is augmented by low external calcium in a G protein-dependent manner (Lu *et al*, 2010; Philippart & Khaliq, 2018), reviewed in (Ren, 2011).). NALCN current is larger in low calcium, in part due to removal of inhibition of NALCN by the G protein-coupled calcium-sensing receptor (Lu *et al*, 2010). Also, in neurons with NALCN current, arresting G protein signaling with GDPBS leads a large inward NALCN current (Philippart & Khaliq, 2018; Lu *et al*, 2010), by removing inhibition by the calcium-sensing receptor. This is opposite of what we observe for GluD1 current. GluD1 current is stimulated by GqPCR activation in a G protein-dependent manner, whereas the tonic GluD1 current is G protein-independent. In our preparation, intracellular GDPBS in the internal did not produce an inward current (control: 12.9±10 pA; 15' dialysis with GDPBS: 39.1±19 pA, p =0.10, Wilcoxon test). This leads us to speculate that there may not be a substantial NALCN current in dorsal raphe serotonin neurons, at least under our recording conditions. To test directly for involvement of NALCN in our preparation, we have also added additional experiments as described below.

The tonic current is gone in the case of GluD1 KO, I realise (eLife 2020 paper). But as it the putative GluD1 leak current examined here seems independent of several other GluD1-specific mechanisms, I think it's wise to give as much explicit information as possible, as to why this possibility (NALCN) is excluded. This is particularly important because in previous work (eLife 2020 paper), the senior author wrote that "a1-adrenergic receptors modulate tonic GluD1R-channel current" even if, in hindsight, this is not really what was meant and isn't the case. One last point, for future work: In an ideal world the GluD1 tonic current that increases in zero calcium would be controlled against KO cells (as was done before for the 2020 eLife paper). That is, the KO should have no calcium dependent NASPM sensitive leak current.

To address any involvement of NALCN, we performed new experiments to evaluate the effect of gadolinium (Gd^{3+}) on the alpha1-adrenergic receptor-stimulated current and tonic current. External Gd^{3+} blocks NALCN efficaciously. While Gd^{3+} is not terrifically selective for NALCN only, it has been used in many studies to determine whether a sodium current is carried

by NALCN channels (Lu *et al*, 2007, 2010, 2009; Chua *et al*, 2020). The results of our new experiments show that Gd^{3+} had no significant effect on the amplitude of the alpha1-AR-EPSC (Expanded view Figure 5A-C). In addition, Gd^{3+} had no significant effect on the magnitude of the tonic NASPM-sensitive current (tonic GluD1 current, Expanded view Figure 5D-E). Therefore, under physiological conditions, NALCN channels do not contribute to the tonic current.

Since decreasing external calcium increases NALCN current, we also tested whether gadolinium affected the tonic NASPM-sensitive current measured in nominally calcium-free external. The data show that the NASPM-sensitive current in 0 mM calcium is not significantly different from the NASPM-sensitive current in 0 mM calcium with Gd^{3+} (Expanded view Figure 5E-F). However, we do note that in Gd^{3+}, the magnitude of the NASPM-sensitive current in 0 mM calcium is no longer statistically larger than in 1.2 mM calcium (Expanded view Figure 5F). One possible interpretation is that in calcium-free conditions there is ~ 5 pA of NALCN current (also assuming that NALCN is blocked by NASPM which has not been tested). Another possible and perhaps more likely explanation is that Gd^{3+} can occupy the same site as Ca^{2+} in GluD1, such that removing Ca^{2+} does not augment tonic GluD1 current to the same degree because Gd^{3+} is present as a blocker. Since we cannot distinguish these possibilities, we have softened the conclusion that tonic GluD1 current is inhibited by physiological level (1.2 mM calcium) – but we believe the data convincingly demonstrate the tonic current is not NALCN.

As for the conclusion in eLife 2020 (the senior author wrote that "a1-adrenergic receptors modulate tonic GluD1R-channel current" even if, in hindsight, this is not really what was meant and isn't the case.) We agree and have adapted our understanding to that “alpha1-AR modulate the same set of GluD1 channels that underly tonic GluD1 current”.

minor
line 361 All other critical reagents were from Sigma-Aldrich.
Why make the distinction? What are non-critical reagents?

We have removed “critical”.

Figure 6 "Tonic GluD1R current is reduced by elevated levels of extracellular calcium."
perhaps it would be better just to say "inhibited by extracellular calcium" ? It's not reduced by elevated calcium in any physiological context, but it is in fact tonically reduced by the normal level of calcium. Perhaps this is just a matter of taste but I think you can be more direct.

We have made the change as suggested.

the labelling in figure 5A is misleading - there was external Ca2+ throughout.

We have corrected this and hope it is now clear.

Fig 1E : is the x-axis reversed and positive in voltage? Surely not. Can you explain what the downward blip is around -30 mV? Calcium channels?

Thank you for pointing this out. This was mislabeled and has been corrected. We are not sure about why the IV deviates from linear around – 30 mV. It may be, as suggested by the reviewer, 1. 'run-down' of calcium current from voltage-gated calcium channels or 2. it may reflect voltage-dependence of GluD1 block by calcium. We note that the IV of the alpha1-EPSC also changes slope around -40 mV (Gantz et al., eLife 2020, Figure 2F). In order to fully understand the downward blip, we need to block calcium current from voltage-gated calcium channels. We have on-going studies on the sensitivity of GluD1 current to divalent ions used to block voltage-gated calcium channels (e.g. cobalt), but this is best suited as a complete other study.

Fig 2B: same problem as in Fig 1E? Missing minus signs?

Thank you for pointing this out. This was mislabeled and has been corrected.

Referee #3:

Summary

This manuscript reports the finding that the tonic GluD1R current is modulated, not by G protein signaling, but instead by extracellular Ca concentration in cells in the dorsal raphe nucleus. This work is significant because it shows that these receptors are modulated by Ca and contribute to resting membrane potential and excitability. Any mechanisms that controls neuronal excitability so strongly should be of interest to the molecular biology community. The finding is robustly documented and alternative hypotheses are tested, including the roles of extracellular D-serine and glycine, that have been shown to gate GluD channels in other preparations. Strengths of this work is the use of adult animals of both sexes. The experiments are focused, the analyses are appropriate overall and the writing is well structured and concise.

Minor comments

Fig 1e, 2b- X-axis should be negative

Thank you for pointing this out. This was mislabeled and has been corrected.

Line 309- Unclear what is meant by the word origin in 'the origin of tonic GluD1R current

We see how the word "origin" may not be the best word choice here as it often refers to a place or time. We think a better word choice is "cause" (cause: the thing that gives rise to an action, phenomenon, or condition, Oxford Dictionary). We have made this change throughout the text.

Chua HC, Wulf M, Weidling C, Rasmussen LP & Pless SA (2020) The NALCN channel complex is voltage sensitive and directly modulated by extracellular calcium. *Sci Adv* 6: eaaz3154

Lu B, Su Y, Das S, Liu J, Xia J & Ren D (2007) The neuronal channel NALCN contributes resting sodium permeability and is required for normal respiratory rhythm. *Cell* 129: 371–383

Lu B, Su Y, Das S, Wang H, Wang Y, Liu J & Ren D (2009) Peptide neurotransmitters activate a cation channel complex of NALCN and UNC-80. *Nature* 457: 741–744

Lu B, Zhang Q, Wang H, Wang Y, Nakayama M & Ren D (2010) Extracellular Calcium Controls Background Current and Neuronal Excitability via an UNC79-UNC80-NALCN Cation Channel Complex. *Neuron* 68: 488–499

Philippart F & Khaliq ZM (2018) Gi/o protein-coupled receptors in dopamine neurons inhibit the sodium leak channel NALCN. *Elife* 7: 1

Ren D (2011) Sodium leak channels in neuronal excitability and rhythmic behaviors. *Neuron* 72: 899–911

Dear Dr. Gantz

Thank you for the submission of your revised manuscript. We have now received the enclosed report from referee 2 who was asked to assess it, and I am happy to say that the referee supports the publication of your study now. Only some more minor editorial requests will need to be addressed before we can proceed with the official acceptance of your manuscript.

- Please add a "Data Availability Section" (DAS) to the end of the Materials and Methods. If you have not deposited any data in public databases, please mention this fact in the DAS.

- Your manuscript has 5 main figures but separate Results and Discussion sections. Please either add one more main figure or combine the Results and Discussion sections to publish your paper as a short report that should not have more than 29.000 characters (including spaces but excluding references and materials and methods).

- Please add up to 5 keywords to the ms file.

- Please remove the author credits from the ms file. We now use CRediT to specify the contributions of each author in the journal submission system. CRediT replaces the author contribution section. Please use the free text box to provide more detailed descriptions, if you wish. See also guide to authors https://www.embopress.org/page/journal/14693178/authorguide#authorshipguidelines.

- Fig EV5A callout is missing, please add.

- The ms sections are in the wrong order, please correct. You can find all info in our guide to authors online.

- I attach to this email a related ms file with comments by our data editors. Please address all comments in the final ms.

I would like to suggest some changes to the title and abstract that needs to be written in present tense. Please let me know whether you agree with the following:

Potentiation of neuronal activity by tonic GluD1 current in brain slices [OK?]

Ion channel function of native delta glutamate receptors (GluDRs) is incompletely understood. Previously, we and others have shown that activation of Gαq protein-coupled receptors (GqPCR) produces a slow inward current carried by GluD1R. GluD1R also carry a tonic cation current of unknown cause. Here, using voltage-clamp electrophysiological recordings from adult mouse brain slices containing the dorsal raphe nucleus, we find no role of on-going G protein-coupled receptor activity in generating or sustaining tonic GluD1R currents. Neither augmentation nor disruption of G protein activity affects tonic GluD1R currents, suggesting that on-going G protein-coupled receptor activity does not give rise to tonic GluD1R currents. Further, the tonic GluD1R current is unaffected by the addition of external glycine or D-serine, which influence GluD2R current at millimolar concentrations. Instead, GqPCR-stimulated and tonic GluD1R currents are regulated by physiological levels of external calcium. In current-clamp recordings, block of GluD1R channels hyperpolarizes the membrane by ~7 mV at subthreshold potentials, reducing excitability. Thus, GluD1R carry a G protein-independent tonic current that contributes to subthreshold neuronal excitation in the dorsal raphe nucleus.

EMBO press papers are accompanied online by A) a short (1-2 sentences) summary of the findings and their significance, B) 2-3 bullet points highlighting key results and C) a synopsis image that is exactly 550 pixels wide and 200-600 pixels high (the height is variable). You can either show a model or key data in the synopsis image. Please note that text needs to be readable at the final size. Please send us this information along with the final manuscript.

I look forward to seeing a final version of your manuscript as soon as possible. Please use this link to submit your revision: https://embor.msubmit.net/cgi-bin/main.plex

Best regards,
Esther

Esther Schnapp, PhD
Senior Editor
EMBO reports

Referee #2:

I have looked over the author's response and the revised manuscript. I think the response is very complete and has improved

what was already a good paper further. The extra experiments with Gd^{3+} are an unexpected bonus and further underline the specificity of this effect. The additions to the text will help the general reader to understand the details of the findings more quickly.

Congratulations to all the authors on this excellent study.

The authors have addressed all minor editorial requests.

Dr. Stephanie Gantz
University of Iowa
Molecular Physiology and Biophysics
51 Newton Rd
BSB 6-429
Iowa City, Iowa 52242
United States

Dear Dr. Gantz,

I am very pleased to accept your manuscript for publication in the next available issue of EMBO reports. Thank you for your contribution to our journal.

At the end of this email I include important information about how to proceed. Please ensure that you take the time to read the information and complete and return the necessary forms to allow us to publish your manuscript as quickly as possible.

As part of the EMBO publication's Transparent Editorial Process, EMBO reports publishes online a Review Process File to accompany accepted manuscripts. As you are aware, this File will be published in conjunction with your paper and will include the referee reports, your point-by-point response and all pertinent correspondence relating to the manuscript.

If you do NOT want this File to be published, please inform the editorial office within 2 days, if you have not done so already, otherwise the File will be published by default [contact: emboreports@embo.org]. If you do opt out, the Review Process File link will point to the following statement: "No Review Process File is available with this article, as the authors have chosen not to make the review process public in this case."

Thank you again for your contribution to EMBO reports and congratulations on a successful publication. Please consider us again in the future for your most exciting work.

Best regards,
Esther

Esther Schnapp, PhD
Senior Editor
EMBO reports

****

THINGS TO DO NOW:

Please note that you will be contacted by Wiley Author Services to complete licensing and payment information. The required 'Page Charges Authorization Form' is available here: https://www.embopress.org/pb-assets/embo-site/er_apc.pdf - please download and complete the form and return to embopressproduction@wiley.com

EMBO Press participates in many Publish and Read agreements that allow authors to publish Open Access with reduced/no publication charges. Check your eligibility: https://authorservices.wiley.com/author-resources/Journal-Authors/open-access/affiliation-policies-payments/index.html

You will receive proofs by e-mail approximately 2-3 weeks after all relevant files have been sent to our Production Office; you should return your corrections within 2 days of receiving the proofs.

Please inform us if there is likely to be any difficulty in reaching you at the above address at that time. Failure to meet our deadlines may result in a delay of publication, or publication without your corrections.

All further communications concerning your paper should quote reference number EMBOR-2023-56801V3 and be addressed to emboreports@wiley.com.

Should you be planning a Press Release on your article, please get in contact with emboreports@wiley.com as early as possible, in order to coordinate publication and release dates.

EMBO Press Author Checklist

Corresponding Author Name: Stephanie C. Gantz	
Journal Submitted to: EMBO Reports	
Manuscript Number: EMBOR-2023-56801	

USEFUL LINKS FOR COMPLETING THIS FORM
The EMBO Journal - Author Guidelines
EMBO Reports - Author Guidelines
Molecular Systems Biology - Author Guidelines
EMBO Molecular Medicine - Author Guidelines

Reporting Checklist for Life Science Articles (updated January 2022)
This checklist is adapted from Materials Design Analysis Reporting (MDAR) Checklist for Authors. MDAR establishes a minimum set of requirements in transparent reporting in the life sciences (see Statement of Task: 10.31222/osf.io/9sm4x). Please follow the journal's guidelines in preparing your manuscript.
Please note that a copy of this checklist will be published alongside your article.

Abridged guidelines for figures
1. Data
The data shown in figures should satisfy the following conditions:
- the data were obtained and processed according to the field's best practice and are presented to reflect the results of the experiments in an accurate and unbiased manner.
- ideally, figure panels should include only measurements that are directly comparable to each other and obtained with the same assay.
- plots include clearly labeled error bars for independent experiments and sample sizes. Unless justified, error bars should not be shown for technical replicates.
- if n<5, the individual data points from each experiment should be plotted. Any statistical test employed should be justified.
- Source Data should be included to report the data underlying figures according to the guidelines set out in the authorship guidelines on Data Presentation.

2. Captions
Each figure caption should contain the following information, for each panel where they are relevant:
- a specification of the experimental system investigated (eg cell line, species name).
- the assay(s) and method(s) used to carry out the reported observations and measurements.
- an explicit mention of the biological and chemical entity(ies) that are being measured.
- an explicit mention of the biological and chemical entity(ies) that are altered/varied/perturbed in a controlled manner.
- the exact sample size (n) for each experimental group/condition, given as a number, not a range;
- a description of the sample collection allowing the reader to understand whether the samples represent technical or biological replicates (including how many animals, litters, cultures, etc.).
- a statement of how many times the experiment shown was independently replicated in the laboratory.
- definitions of statistical methods and measures:
  - common tests, such as t-test (please specify whether paired vs. unpaired), simple χ2 tests, Wilcoxon and Mann-Whitney tests, can be unambiguously identified by name only, but more complex techniques should be described in the methods section;
  - are tests one-sided or two-sided?
  - are there adjustments for multiple comparisons?
  - exact statistical test results, e.g., P values = x but not P values < x;
  - definition of 'center values' as median or average;
  - definition of error bars as s.d. or s.e.m.

Please complete ALL of the questions below.
Select "Not Applicable" only when the requested information is not relevant for your study.

Materials

Newly Created Materials	Information included in the manuscript?	In which section is the information available? (Reagents and Tools Table, Materials and Methods, Figures, Data Availability Section)
New materials and reagents need to be available; do any restrictions apply?	Not Applicable	

Antibodies	Information included in the manuscript?	In which section is the information available? (Reagents and Tools Table, Materials and Methods, Figures, Data Availability Section)
For **antibodies** provide the following information:<br>- Commercial antibodies: RRID (if possible) or supplier name, catalogue number and or/clone number<br>- Non-commercial: RRID or citation	Not Applicable	

DNA and RNA sequences	Information included in the manuscript?	In which section is the information available? (Reagents and Tools Table, Materials and Methods, Figures, Data Availability Section)
**Short novel DNA or RNA including primers, probes**: provide the sequences.	Not Applicable	

Cell materials	Information included in the manuscript?	In which section is the information available? (Reagents and Tools Table, Materials and Methods, Figures, Data Availability Section)
**Cell lines:** Provide species information, strain. Provide accession number in repository **OR** supplier name, catalog number, clone number, and/**OR** RRID.	Not Applicable	
**Primary cultures:** Provide species, strain, sex of origin, genetic modification status.	Not Applicable	
Report if the cell lines were recently **authenticated** (e.g., by STR profiling) and tested for mycoplasma contamination.	Not Applicable	

Experimental animals	Information included in the manuscript?	In which section is the information available? (Reagents and Tools Table, Materials and Methods, Figures, Data Availability Section)
**Laboratory animals or Model organisms:** Provide species, strain, sex, age, genetic modification status. Provide accession number in repository **OR** supplier name, catalog number, clone number, **OR** RRID.	Yes	Materials and Methods
**Animal observed in or captured from the field:** Provide species, sex, and age where possible.	Not Applicable	
Please detail **housing and husbandry conditions**.	Yes	Materials and Methods

Plants and microbes	Information included in the manuscript?	In which section is the information available? (Reagents and Tools Table, Materials and Methods, Figures, Data Availability Section)
**Plants:** provide species and strain, ecotype and cultivar where relevant, unique accession number if available, and source (including location for collected wild specimens).	Not Applicable	
**Microbes:** provide species and strain, unique accession number if available, and source.	Not Applicable	

Human research participants	Information included in the manuscript?	In which section is the information available? (Reagents and Tools Table, Materials and Methods, Figures, Data Availability Section)
If collected and within the bounds of privacy constraints report on age, sex and gender or ethnicity for all study participants.	Not Applicable	

Core facilities	Information included in the manuscript?	In which section is the information available? (Reagents and Tools Table, Materials and Methods, Figures, Data Availability Section)
If your work benefited from core facilities, was their service mentioned in the acknowledgments section?	Not Applicable	

Design

Study protocol	Information included in the manuscript?	In which section is the information available? (Reagents and Tools Table, Materials and Methods, Figures, Data Availability Section)
If study protocol has been **pre-registered, provide DOI in the manuscript**. For clinical trials, provide the trial registration number **OR** cite DOI.	Not Applicable	
Report the **clinical trial registration number** (at ClinicalTrials.gov or equivalent), where applicable.	Not Applicable	

Laboratory protocol	Information included in the manuscript?	In which section is the information available? (Reagents and Tools Table, Materials and Methods, Figures, Data Availability Section)
Provide DOI OR other citation details if **external detailed step-by-step protocols** are available.	Not Applicable	

Experimental study design and statistics	Information included in the manuscript?	In which section is the information available? (Reagents and Tools Table, Materials and Methods, Figures, Data Availability Section)
Include a statement about **sample size** estimate even if no statistical methods were used.	Yes	Materials and Methods
Were any steps taken to minimize the effects of subjective bias when allocating animals/samples to treatment (e.g. **randomization procedure**)? If yes, have they been described?	Not Applicable	
Include a statement about **blinding** even if no blinding was done.	Yes	Materials and Methods
Describe **inclusion/exclusion criteria** if samples or animals were excluded from the analysis. Were the criteria pre-established?<br><br>If sample or data points were omitted from analysis, report if this was due to attrition or intentional exclusion and provide justification.	Not Applicable	
For every figure, are **statistical tests** justified as appropriate? Do the data meet the assumptions of the tests (e.g., normal distribution)? Describe any methods used to assess it. Is there an estimate of variation within each group of data? Is the variance similar between the groups that are being statistically compared?	Yes	Materials and Methods

Sample definition and in-laboratory replication	Information included in the manuscript?	In which section is the information available? (Reagents and Tools Table, Materials and Methods, Figures, Data Availability Section)
In the figure legends: state number of times the experiment was **replicated** in laboratory.	Yes	Figure legend
In the figure legends: define whether data describe **technical or biological replicates**.	Yes	materials and methods

Ethics

Ethics	Information included in the manuscript?	In which section is the information available? (Reagents and Tools Table, Materials and Methods, Figures, Data Availability Section)
Studies involving **human participants**: State details of **authority granting ethics approval** (IRB or equivalent committee(s), provide reference number for approval.	Not Applicable	
Studies involving **human participants**: Include a statement confirming that **informed consent** was obtained from all subjects and that the experiments conformed to the principles set out in the WMA Declaration of Helsinki and the Department of Health and Human Services Belmont Report.	Not Applicable	
Studies involving **human participants:** For publication of **patient photos**, include a statement confirming that consent to publish was obtained.	Not Applicable	
Studies involving experimental **animals**: State details of **authority granting ethics approval** (IRB or equivalent committee(s), provide reference number for approval. Include a statement of compliance with ethical regulations.	Yes	Materials and Methods
Studies involving **specimen and field samples:** State if relevant **permits** obtained, provide details of authority approving study; if none were required, explain why.	Not Applicable	

Dual Use Research of Concern (DURC)	Information included in the manuscript?	In which section is the information available? (Reagents and Tools Table, Materials and Methods, Figures, Data Availability Section)
Could your study fall under dual use research restrictions? Please check biosecurity documents and list of **select agents and toxins** (CDC): https://www.selectagents.gov/sat/list.htm	Not Applicable	
If you used a select agent, is the security level of the lab appropriate and reported in the manuscript?	Not Applicable	
If a study is subject to dual use research of concern regulations, is the name of the **authority granting approval and reference number** for the regulatory approval provided in the manuscript?	Not Applicable	

Reporting

The MDAR framework recommends adoption of discipline-specific guidelines, established and endorsed through community initiatives. Journals have their own policy about requiring specific guidelines and recommendations to complement MDAR.

Adherence to community standards	Information included in the manuscript?	In which section is the information available? (Reagents and Tools Table, Materials and Methods, Figures, Data Availability Section)
State if relevant guidelines or checklists (e.g., **ICMJE, MIBBI, ARRIVE, PRISMA**) have been followed or provided.	Not Applicable	
For **tumor marker prognostic studies,** we recommend that you follow the **REMARK** reporting guidelines (see link list at top right). See author guidelines, under 'Reporting Guidelines'. Please confirm you have followed these guidelines.	Not Applicable	
For **phase II and III randomized controlled trials**, please refer to the **CONSORT** flow diagram (see link list at top right) and submit the CONSORT checklist (see link list at top right) with your submission. See author guidelines, under 'Reporting Guidelines'. Please confirm you have submitted this list.	Not Applicable	

Data Availability

Data availability	Information included in the manuscript?	In which section is the information available? (Reagents and Tools Table, Materials and Methods, Figures, Data Availability Section)
Have **primary datasets** been deposited according to the journal's guidelines (see 'Data Deposition' section) and the respective accession numbers provided in the Data Availability Section?	Not Applicable	Available on request
Were **human clinical and genomic datasets** deposited in a public access-controlled repository in accordance to ethical obligations to the patients and to the applicable consent agreement?	Not Applicable	
Are **computational models** that are central and integral to a study available without restrictions in a machine-readable form? Were the relevant accession numbers or links provided?	Not Applicable	
If publicly available data were reused, provide the respective **data citations in the reference list**.	Not Applicable	
